# Structure-Guided Design of Selective Epac1 and Epac2 Agonists

**DOI:** 10.1371/journal.pbio.1002038

**Published:** 2015-01-20

**Authors:** Frank Schwede, Daniela Bertinetti, Carianne N. Langerijs, Michael A. Hadders, Hans Wienk, Johanne H. Ellenbroek, Eelco J. P. de Koning, Johannes L. Bos, Friedrich W. Herberg, Hans-Gottfried Genieser, Richard A. J. Janssen, Holger Rehmann

**Affiliations:** 1 BIOLOG Life Science Institute, Bremen, Germany; 2 Department of Biochemistry, University of Kassel, Kassel, Germany; 3 Galapagos BV, CR Leiden, The Netherlands; 4 Department of Chemistry, Laboratory of Crystal and Structural Chemistry, Bijvoet Center for Biomolecular Research, Utrecht University, Utrecht, The Netherlands; 5 Department of Chemistry, NMR Spectroscopy, Bijvoet Center for Biomolecular Research, Utrecht University, Utrecht, The Netherlands; 6 Department of Nephrology, Leiden University Medical Center, Leiden, The Netherlands; 7 Hubrecht Institute/KNAW and University Medical Center Utrecht, Utrecht, The Netherlands; 8 Molecular Cancer Research and Cancer Genomics Netherlands, Center for Molecular Medicine, UMC Utrecht, Utrecht, The Netherlands; Brandeis University, UNITED STATES

## Abstract

The second messenger cAMP is known to augment glucose-induced insulin secretion. However, its downstream targets in pancreatic β-cells have not been unequivocally determined. Therefore, we designed cAMP analogues by a structure-guided approach that act as Epac2-selective agonists both *in vitro* and *in vivo*. These analogues activate Epac2 about two orders of magnitude more potently than cAMP. The high potency arises from increased affinity as well as increased maximal activation. Crystallographic studies demonstrate that this is due to unique interactions. At least one of the Epac2-specific agonists, Sp-8-BnT-cAMPS (S-220), enhances glucose-induced insulin secretion in human pancreatic cells. Selective targeting of Epac2 is thus proven possible and may be an option in diabetes treatment.

## Author Summary

cAMP is a small molecule produced by cells that activates proteins involved in a wide range of biological processes, including olfaction, pacemaker activity, regulation of gene expression, insulin secretion, and many others. In the case of insulin secretion, cAMP seems to impinge on different stages of the signalling cascade to regulate secretory activity in pancreatic β-cells. Here we have developed a chemically modified version of cAMP that specifically only activates Epac2, one of the cAMP-responsive proteins in this cascade. Furthermore, our cAMP analogue activates Epac2 more potently than cAMP itself does. We have determined several crystal structures of Epac2 in complex with cAMP analogues to help us explain the molecular basis of the observed selectivity and the strong activation potential. In addition, we were able to show that the analogue is able to potentiate glucose-induced secretion of insulin from human pancreatic islets. The principal challenge during this study was identifying and understanding small differences in the cAMP-binding domains of cAMP-regulated proteins and matching these differences with suitable modifications of the cAMP molecule.

## Abstract 3

A newly developed analogue of cAMP that selectively activates Epac2 can potentiate glucose-induced insulin secretion from human pancreatic β-cells.

## Introduction

Food intake enhances insulin secretion from pancreatic β-cells in two ways. First, an elevated glucose concentration in the blood increases the availability of glucose and thus the rate of ATP formation by glycolysis in β-cells. The increased cellular ATP concentration causes the closure of ATP sensitive K^+^-channels and thereby depolarization of the cell [1–3]. In turn, voltage dependent Ca^2+^ channels open and the raise in the cellular Ca^2+^ concentration causes the fusion of insulin granules with the plasma membrane and thus insulin secretion. Second, this glucose-induced insulin secretion is further enhanced by the incretin hormones glucose-dependent insulinotropic peptide/gastric inhibitory peptide (GIP) and glucagon-like peptide-1 (GLP-1), which are released upon food intake by the gut. The receptors for GIP and GLP-1 are coupled to adenylyl cyclase, which mediates the formation of the second messenger cAMP in β-cells [4,5].

**Figure 1:**
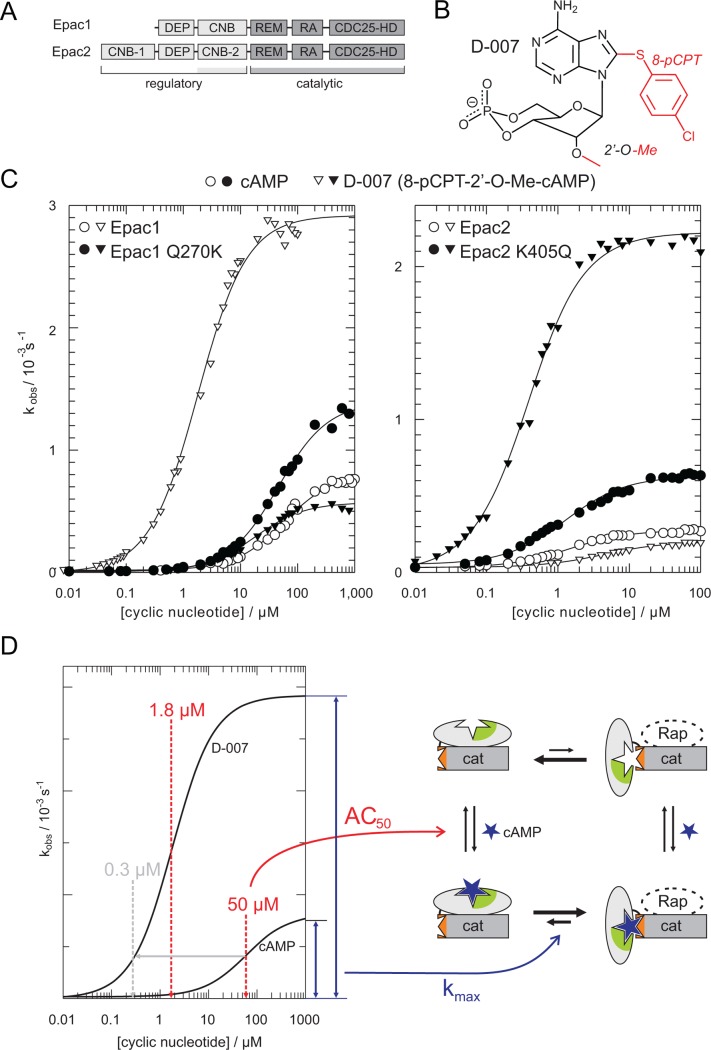
D-007 is an efficient activator of Epac1 but not of Epac2. (A) Domain organization of Epac1 and Epac2. Only the second CNB domain of Epac2 is involved in the regulation process. DEP, Dishevelled, Egl-10 and Pleckstrin; REM, Ras exchange motif; RA, Ras Association; CDC25-HD, CDC25-homology domain. (B) Chemical structure of D-007. (C) Comparison of cyclic nucleotide induced activity for Epac1, Epac1^Q270K^, Epac2^Δ280^, and Epac2^Δ280,K405Q^. Position 270 of Epac1 corresponds to position 405 in Epac2. Plot data can be found in S1 Data. (D) Illustration of data interpretation. Epac exists in equilibrium between an inactive and active conformation (right). The AC_50_ is a measure for the affinity of the cAMP analogue and the k_max_ for the extent to what the equilibrium is shifted to the active conformation. The concentration of an analogue required to reach the same activity as induced by cAMP at its AC_50_ is defined as the activation potential (indicated by grey lines and arrows).

Generally cAMP acts via cAMP-dependent protein kinase (PKA), cyclic nucleotide regulated ion channels, and exchange protein activated by cAMP (Epac) proteins by its direct interaction with highly related cyclic nucleotide binding (CNB) domains [6,7]. Epac proteins are guanine nucleotide exchange factors for the small G-protein Rap [8,9]. Epac1 and Epac2 contain one and two N-terminal CNB domains, respectively (Fig. 1A). Only the second CNB domain of Epac2 controls the proteins exchange activity [10]. The CNB domain blocks the access of Rap to the C-terminal catalytic site in the inactive state [11] and swings away upon activation [10]. The active and the inactive conformation are in equilibrium in the ligand-free and the ligand bound state, and the binding of agonists shifts the equilibrium to various extents to the active conformation [12,13]. The activity induced by an agonist under saturating conditions is termed maximal activity (k_max_) and is a measure to what extent the equilibrium is shifted to the active conformation (Fig. 1D). The natural agonist cAMP shifts the equilibrium only partially. It was shown for Epac1 that some cAMP analogues shift the equilibrium up to three times more effectively than cAMP [13].

The pathways by which cAMP augments glucose induced insulin secretion are not fully understood [5]. A function of PKA was demonstrated by selective inhibition of PKA [14]; however, other studies reported only a partial effect [15–17] or even no effect of PKA inhibition [18]. Epac2 was linked to insulin secretion first because of its ability to interact with ATP sensitive K^+^-channels and with Rim [19,20]. Furthermore it was proposed that Epac contributes to the release of insulin granules by increasing the cellular Ca^2+^ concentration [21–23], by increasing the granule density at the plasma membrane [24], and by promoting the acidification of the granules [25]. Likely, these effects are mainly mediated by Epac2, as in β-cells Epac1 is expressed at much lower levels than Epac2 [26]. Thus cAMP seems to act at various levels on the process of exocytosis and to act via PKA and Epac-dependent pathways. In agreement with this observation, a recent electrophysiological study has demonstrated that PKA- and Epac-dependent processes enhance the Ca^2+^-sensitivity and the rate of exocytosis, respectively [27].

Two classes of anti-diabetic drugs are suggested to impinge on Epac2. First, the potency of sulfonylureas, which act as blockers of the ATP sensitive K^+^-channel in β-cells, is reduced in Epac2^−/−^ mice [28]. Even though a direct binding of sulfonylureas to Epac2 was suggested [28], this interaction could not be confirmed in independent studies [29,30]. Second, exenatide and liraglutide act as GLP-1 receptor agonists and therefore induce formation of cAMP in β-cells. The GLP-1 mediated enhancement of insulin secretion and the blood-pressure lowering effect of liraglutide are mediated at least partially by Epac2 [19,22–25,31].

Direct targeting of Epac2 may be an option for diabetes treatment. Putative rare adverse events of exenatide and liraglutide [32,33] may be circumvented by direct targeting of Epac2, in particular since Epac2 is mainly expressed in pancreatic β-cells [26]. Exenatide and liraglutide are applied by subcutaneous injection and orally applicable alternatives would ease treatment. For the exploration of Epac2 as drug target a better understanding of its signaling is key, and an Epac2 selective agonist would be a valuable tool for such an analysis and for a proof of concept. Therefore, in this study, we aimed for the design of an Epac2 selective cAMP analogue.

## Results

### Epac1 Is Distinct from Epac2

**Figure 2:**
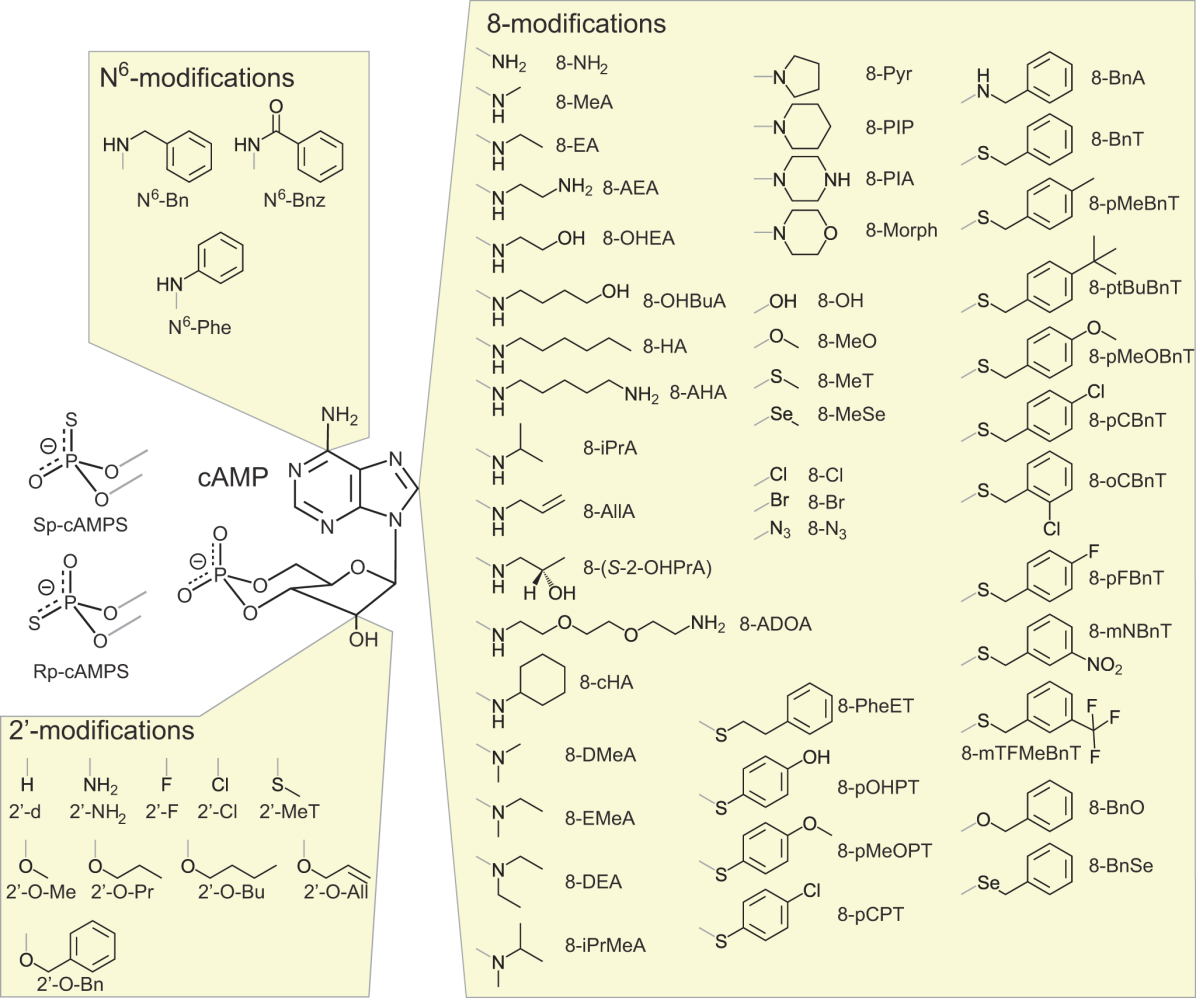
Chemical structures of cAMP analogues. cAMP was modified in the cyclic phosphate and at N^6^-, 2′-, and 8-positions. Modifications are labelled with the nomenclature used for the abbreviated names of the cAMP analogues as used in Table 1. Structures of analogues with two or more modifications can be constructed based on the abbreviated names given in Table 1 and S1 Table. S1 Table gives the full names.

In biological research the cAMP analogue D-007 is used as a selective activator of Epac proteins, since it does not act on PKA or cyclic nucleotide regulated ion channels (for chemical structure and abbreviation of cAMP analogues see Fig. 2 and S1 Table) [34,35]. D-007 is modified with a *para*-chlorophenylthio (pCPT) substituent at the 8-position and an O-methyl substituent (O-Me) at the 2′-position (Fig. 1B). D-007 efficiently activates Epac1 with an AC_50_ of 1.8 μM and relative maximal activity (k_max_) of 3.3. D-007 is thus a stronger agonist than cAMP (AC_50_ = 50 μM; k_max_ = 1) (Fig. 1C and 1D; Table 1) [13]. L-026, which carries only the 8-pCPT-modification, activates Epac1 with an AC_50_ of 0.9 μM and a relative k_max_ of 0.5. Z-004, which carries only the 2′-O-Me-modification, has a reduced affinity but a higher maximal activity than cAMP (AC_50_ > 100 μM; k_max_ > 2) (Table 1) [13]. Thus, the 8-pCPT-modification in D-007 and L-026 is responsible for the high affinity, and the 2′-O-Me-modification in D-007 and Z-004 is responsible for the high maximal activity.

**Table 1:** Activation constants for Epac.

**Modification**	**Identifier**	**Analogue^a^**	**Epac2^Δ280^**		**Epac2^fl^**		**Epac1**	
			**AC_50_/μM**	**Rel. k_max_**	**AC_50_/μM**	**Rel. k_max_**	**AC_50_/μM**	**Rel. k_max_**
						
Unmodified		cAMP	1.8^b^	1.0^b^	20^b^	1.0^b^	45^c^	1.0^c^
2′-modification	Z-001	2′-dcAMP	70	1.4			>1,000^c^	
	**Z-002**	**2′-F-cAMP**	25	1.0			1,500	1.2?
	**Z-003**	**2′-NH_2_-cAMP**	9.0	0.9			80	0.8
	Z-004	2′-O-Me-cAMP	32	1.0			>100^c^	>2^c^
	Z-005	2′-O-Pr-cAMP	70	0.5				
	Z-006	2′-O-Bu-cAMP	120	1.0				
	**Z-007**	**2′-O-All-cAMP**	70	1.0				
	**Z-008**	**2′-O-Bn-cAMP**	35	0.4			75	1.5
								
8-modification	L-001	8-Cl-cAMP	0.3	0.8			6.9^c^	0.5^c^
	L-002	8-Br-cAMP	0.4	0.9			6.2^c^	0.5^c^
	L-003	8-NH_2_-cAMP	0.2	0.7				
	L-004	8-N_3_-cAMP	0.2	0.7				
	L-005	8-MeA-cAMP	0.6	3.3			19^c^	0.5^c^
	L-006	8-AEA-cAMP	1.6	0.5				
	L-007	8-OHEA-cAMP	2.6	1.0				
	L-008	8-OHBuA-cAMP	2.6	1.6				
	L-009	8-HA-cAMP	2.5	2.0				
	L-010	8-AHA-cAMP	1.2	1.8				
	**L-011**	**8-AllA-cAMP**	1.9	1.5				
	L-012	8-(*S*-2-OHPrA)-cAMP	8.0	0.5				
	**L-013**	**8-cHA-cAMP**	4.7	0.3			50	0.3
	L-014	8-BnA-cAMP	1.9	1.5			30	0.2
	L-015	8-DMeA-cAMP	1.4	3.6				
	L-016	8-DEA-cAMP	9.0	5.1				
	L-017	8-Pyr-cAMP	3.9	4.4				
	L-018	8-PIP-cAMP	13	3.8			130^c^	0.9^c^
	L-019	8-PIA-cAMP	19	3.8				
	**L-020**	**8-Morph-cAMP**	18	3.8				
	L-021	8-OH-cAMP	0.6	0.4			26^c^	0.5^c^
	L-022	8-MeO-cAMP	0.6	2.0				
	L-023	8-BnO-cAMP	1.3	1.6				
	L-024	8-MeT-cAMP	0.14	2.0				
	L-025	8-BnT-cAMP	0.08	2.6			3.6	0.2
	L-026	8-pCPT-cAMP	0.08	0.5			0.9^c^	0.5^c^
	**L-027**	**8-MeSe-cAMP**	0.18	1.5				
	**L-028**	**8-BnSe-cAMP**	0.03	1.7				
								
N6-modification	N-001	N^6^-Bn-cAMP	1.1	0.3				
	N-002	N^6^-Bnz-cAMP	2.9	0.1				
	N-003	N^6^-Phe-cAMP	0.3	0.6				
								
2′-modification + 8-modification	**D-001**	**8-MeA-2′-Cl-cAMP**	45	2.2			330	0.6?
	**D-002**	**8,2′-DMeT-cAMP**	2.6	2.0	110	2.4	17	1.4
	**D-003**	**8-Br-2′-Cl-cAMP**	11	0.9			260	0.5?
	**D-004**	**8-Br-2′-F-cAMP**	14	1.5			100	0.4
	D-005	8-Br-2′-O-Me-cAMP	8.0	1.6			35	0.9
	**D-006**	**8,2′-DCl-cAMP**	14	0.7				
	D-007	8-pCPT-2′-O-Me-cAMP	3.5	0.8	16	0.4	1.8^c^	3.3^c^
	D-008	8-pOHPT-2′-O-Me-cAMP	1.5	2.2			1.0	3.1
	D-009	8-pMeOPT-2′-O-Me-cAMP	2.8	0.5			2.3	2.7
	D-010	8-OH-2′-O-Me-cAMP	6.8	0.5				
	**D-011**	**8-BnT-2′-F-cAMP**	2.9	1.6				
	**D-012**	**8-BnSe-2′-O-Bn-cAMP**	4.6	1.0			38	0.9
								
**D-013**	**8-BnSe-2′-O-Me-cAMP**	3.6	2.3				
								
Sp-Phosphorothioate	S-000	Sp-cAMPS	2.8	2.5			90^c^	1.4^c^
								
Phosphorothioate with 8- and/or 2′-modification	S-010	Sp-8-Br-cAMPS	0.4	3.5	8.0	3.6	16	1.0
	S-011	Sp-8-Br-2′dcAMPS	9.0	0.8				
	**S-012**	**Sp-8-Br-2′-F-cAMPS**	9.8	1.2			75	0.08
	**S-013**	**Sp-8-Br-2′-Cl-cAMPS**	8.0	0.5			110	0.1
	S-014	Sp-8-Br-2′-O-Me-cAMPS	13	2.0	>80			
	S-020	Sp-8-Cl-cAMPS	0.4	3.8				
	**S-021**	**Sp-8,2′-Cl-cAMPS**	12	0.5				
	**S-030**	**Sp-8-MeA-cAMPS**	0.7	8.4	10	7.7	30	1.4
	**S-031**	**Sp-8-MeA-2′-dcAMPS**	40	1.7			320	0.1
	**S-032**	**Sp-8-MeA-2′-F-cAMPS**	9.5	3.9				
	**S-033**	**Sp-8-MeA-2′-Cl-cAMPS**	14	0.7			120	0.1
	**S-034**	**Sp-8-MeA-2′-O-Me-cAMPS**	20	0.7				
	**S-040**	**Sp-8-EA-cAMPS**	1.9	5.4				
	**S-050**	**Sp-8-iPrA-cAMPS**	9.0	1.4				
	S-060	Sp-8-ADOA-cAMPS	6.2	2.1				
	**S-070**	**Sp-8-DMeA-cAMPS**	2.1	9.3				
	**S-080**	**Sp-8-EMeA-cAMPS**	1.8	7.6				
	**S-090**	**Sp-8-DEA-cAMPS**	10	4.1				
	**S-100**	**Sp-8-iPrMeA-cAMPS**	7	4.5				
	S-110	Sp-8-PIP-cAMPS	9.5	3.7				
	S-120	Sp-8-PIA-cAMPS	14	2.3				
	**S-130**	**Sp-8-OH-cAMPS**	1.3	1.4			80	0.6
	**S-140**	**Sp-8-MeO-cAMPS**	0.7	7.3				
	**S-150**	**Sp-8-BnO-cAMPS**	1.3	4.8				
	**S-160**	**Sp-8-MeT-cAMPS**	0.3	7.4				
	**S-170**	**Sp-8-MeT-2′-F-cAMPS**	6.9	2.8				
	**S-180**	**Sp-8-MeT-2′-O-Me-cAMPS**	3.7	3.2				
	**S-190**	**Sp-8-PheET-cAMPS**	0.14	0.4			6.0	0.2
	**S-200**	**Sp-8-pOHPT-cAMPS**	0.07	1.2				
	**S-201**	**Sp-8-pOHPT-2′-F-cAMPS**	1.6	1.0				
	**S-202**	**Sp-8-pOHPT-2′-O-Me-cAMPS**	1.6	1.4				
	S-210	Sp-8-pCPT-cAMPS	0.13	0.7			2.2	0.5
	S-211	Sp-8-pCPT-2′-O-Me-cAMPS	1.4	0.4			7.3	1.9
	**S-220**	**Sp-8-BnT-cAMPS**	0.1	7.7	2.1	6.6	13	0.3
	**S-221**	**Sp-8-BnT-2′-dcAMPS**	10	1.5			280	0.06
	**S-222**	**Sp-8-BnT-2′-F-cAMPS**	4.3	4.2	15	1.8	60	0.09
	**S-223**	**Sp-8-BnT-2′-O-Me-cAMPS**	1.5	4.3	14	2.6	30	0.2
	**S-230**	**Sp-8-pMeBnT-cAMPS**	0.07	6.4				
	**S-240**	**Sp-8-ptBuBnT-cAMPS**	0.10	5.0				
	**S-250**	**Sp-8-pMeOBnT-cAMPS**	0.11	5.4				
	**S-260**	**Sp-8-pCBnT-cAMPS**	0.08	4.3				
	**S-270**	**Sp-8-oCBnT-cAMPS**	0.27	3.1			16	0.2
	**S-280**	**Sp-8-pFBnT-cAMPS**	0.11	4.9				
	**S-290**	**Sp-8-mNBnT-cAMPS**	0.16	4.3				
	**S-300**	**Sp-8-mTFMeBnT-cAMPS**	0.14	5.0				
	S-400	Sp-5,6-DCl-cBIMPS	0.9	0.8			14	1.0
								
Rp-Phospohorothiate	R-000	Rp-cAMPS	10	0.2				

^a^Analogues printed in bold were synthesised for the first time, to our knowledge, during this study. For a visualisation of the chemical structures see Fig. 2. Full chemical names are given in S1 Table.

^b^See also [11].

^c^See also [13].

We extended this analysis to Epac2 by using an N-terminal truncated version that lacks the “irrelevant” first CNB domain. This construct is activated by cAMP with an AC_50_ of 1.8 μM and a relative k_max_ of 1. Interestingly, Z-004 has the same k_max_ as cAMP. Thus the 2′-O-Me-modification does not improve the maximal activity of Epac2 contrary to the maximal activity of Epac1. Consequently, D-007 activates Epac2 with an AC_50_ of 3.5 μM and a relative k_max_ of 0.8 (Fig. 1C; Table 1). The maximal activity is thus reduced and not enhanced if compared to cAMP and therefore D-007 is a poor activator of Epac2.

The 2′-OH group of cAMP, which is replaced by the O-Me group in D-007 and Z-004, is known to be involved in critical interactions with CNB domains. In PKA and cyclic nucleotide regulated ion channels, the 2′-OH group of cAMP forms a hydrogen-bond with the side chain of a conserved glutamic acid [36,37]. Instead of glutamic acid, Epac1 contains a glutamine (Gln^270^) and Epac2 contains a lysine (Lys^405^). The consequences of these differences were analyzed by site directed mutagenesis and activity assays. Epac1^Q270K^, like Epac2, is poorly activated by D-007, whereas Epac2^K405Q^ is efficiently activated by D-007 (Fig. 1C). Thus, indeed a single amino acid determines the differential response of Epac1 and Epac2 to D-007.

**Figure 3:**
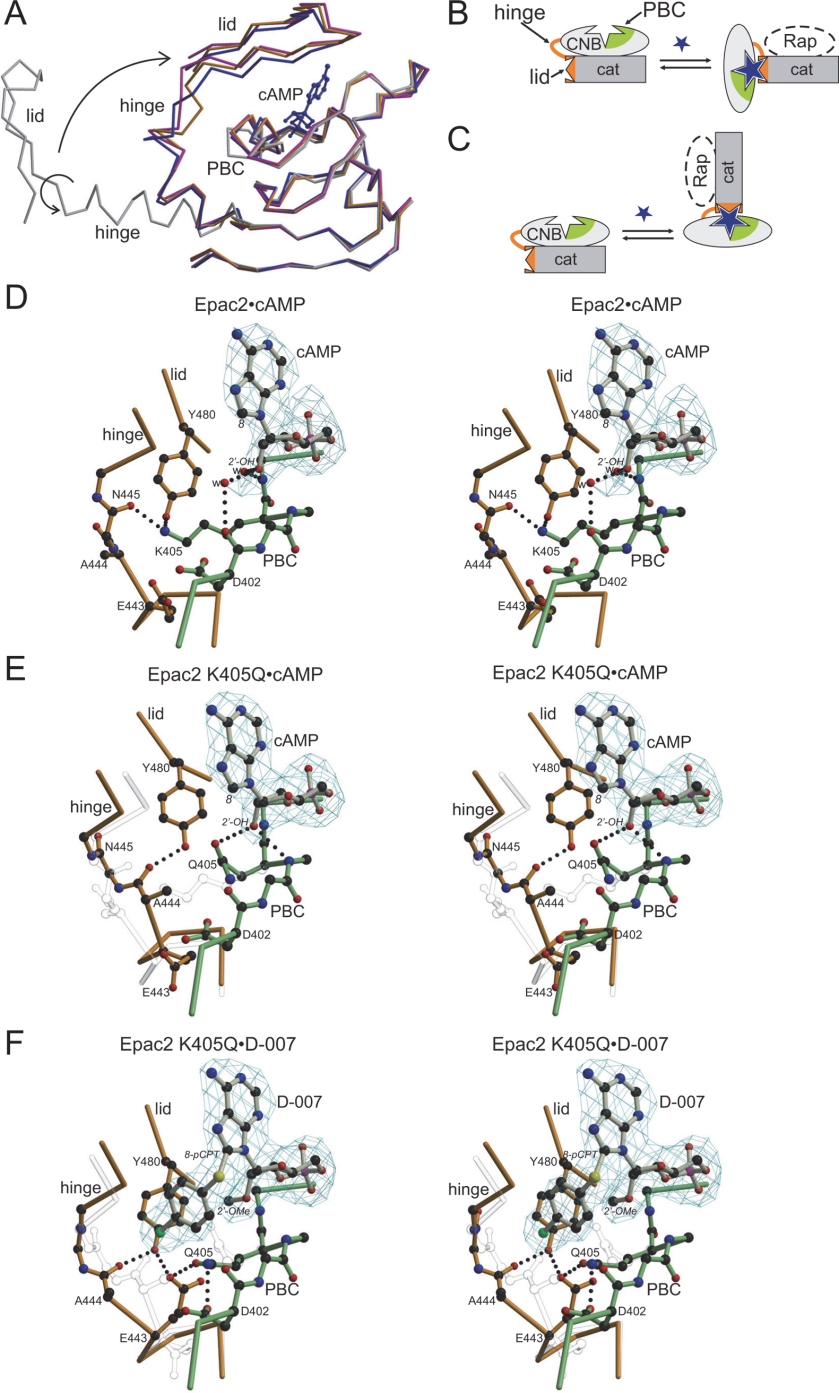
Structural basis for efficient activation of Epac1 by D-007. (A) Superposition of the CNB domains of Epac2 in the absence of cAMP (grey) and bound to cAMP (blue), and of Epac2^K405Q^ bound to cAMP (orange) and D-007 (magenta). Binding of cAMP induces conformational changes in the phosphate binding cassette (PBC) which allow the hinge to move and the lid to flip over the CNB site, where the lid it is anchored by interactions with the base of the cyclic nucleotide [10]. cAMP originates from Epac2•cAMP•Rap. Arrows illustrate the transition from the inactive to the active conformation. (B) Schematic representation of the transition of Epac to the active conformation whereby the conformational change is described as a rigid body movement of the CNB domain. (C) Alternative view on the transition in which the CNB domain is kept fixed. This point of view is adapted in (A and D–F). (D–F) Stereo view of the cAMP binding site with bound cAMP or D-007 with *fofc* density calculated in the absence of the nucleotides. The interaction networks of Lys^405^ and Gln^405^, respectively, are shown. Residues from the PBC are shown in green, and those from the hinge and the lid in orange. The 2′- and the 8-position of the cyclic nucleotides are indicated in italics. Amino acids are given by single letter code; w, water. Epac2•cAMP•Rap (D) Epac2^K405Q^•cAMP•Rap with elements from Epac2•cAMP•Rap in transparent grey (E) and Epac2^K405Q^•D-007•Rap with elements from Epac2^K405Q^•cAMP•Rap in transparent grey (F). (D–F) are in the same orientation based on a super-position of the CNB domains.

The structures of the complexes Epac2•cAMP•Rap, Epac2^K405Q^•cAMP•Rap and Epac2^K405Q^•D-007•Rap were determined, whereby the K405Q mutant served as a model of Epac1 (Fig. 3; Table 2). The structure of Epac2•cAMP•Rap is virtually identical to the previously determined structure of Epac2•S-000•Rap [10], where S-000 had been used as an unnecessary precaution because of its improved hydrolysis resistance. Overall all three newly determined structures are highly similar, but are distinguished by unique conformations in a short region called the hinge (Fig. 3A). The hinge rearranges upon cAMP binding and is responsible for the rigid body movement of the CNB domain, which liberates the catalytic site (Fig. 3B and 3C). In wild-type Epac2•cAMP•Rap, Lys^405^ points away from the cAMP molecule and interacts with the hinge (Fig. 3D). In Epac2^K405Q^•cAMP•Rap, Gln^405^ is turned towards cAMP and forms a hydrogen bond with the 2′-OH-group. The position of Gln^405^ allows the hinge to alter its conformation, which results in a different hydrogen bond network near Tyr^480^ (Fig. 3E). The conformation of the hinge in Epac2^K405Q^•cAMP•Rap would result in clashes with Lys^405^ in the wild-type protein (Fig. 3E). Overall, this situation favors the active conformation, as Epac2^K405Q^ displays a higher maximal activity upon binding of cAMP than Epac2 (Fig. 1C).

**Table 2:** Data collection and refinement statistic.

**Protein^a^ Nucleotide pdb Entry**	**wt cAMP (4MGI)**	**K450Q cAMP (4MGK)**	**K405Q D-007 (4MGY)**	**wt S-220 (4MGZ)**	**wt S-280 (4MH0)**
**Data collection**					
Space group	I2_1_2_1_2_1_	I2_1_2_1_2_1_	I2_1_2_1_2_1_	I2_1_2_1_2_1_	I2_1_2_1_2_1_
Cell dimensions (Å)	a = 125.3	a = 125.0	a = 125.6	a = 125.2	a = 125.8
	b = 148.5	b = 144.6	b = 145.2	b = 147.1	b = 148.7
	c = 224.7	c = 227.8	c = 230.0	c = 226.7	c = 225.2
Wavelength (Å)	1.07225	0.972	0.87260	1.07225	1.07225
Resolution range (Å)^b^	40–2.8 (2.9–2.8)	20–2.7 (2.8–2.7)	40–2.6 (2.7–2.6)	40–3.0 (3.1–3.0)	40–2.4 (2.5–2.4)
Number of reflections	199,618	221,303	240,641	160,040	298,856
Number unique reflections	51,460	56,554	64,657	41,504	81,301
Completeness (%)^b^	99.2 (98.6)	99.3 (99.8)	99.7 (99.9)	98.2 (99.6)	98.5 (97.3)
Redundancy	3.9 (3.9)	4.1 (4.2)	3.7 (3.8)	3.9 (3.9)	3.7 (3.4)
I/σ^b^	16.4 (3.45)	19.7 (3.03)	14.1 (2.92)	16.4 (4.6)	13.0 (2.21)
R_meas_ (%)^b^	9.2 (59.5)	6.8 (54.2)	9.2 (55.1)	10.2 (51.1)	8.2 (75.7)
**Refinement**					
R_cryst_ (%)	24.9	24.7	25.2	23.8	26.6
R_free_ (%)^c^	27.1	26.4	27.0	26.4	28.2
Reflection omitted (%)	4.8	4.8	4.9	4.8	4.9
Number of atoms	6384	6402	6505	6405	6532
Protein	6238	6238	6238	6280	6238
Ligands/ions	49	27	36	58	67
Water	97	137	231	67	229
Average B factor (Å^2^)	53.1	53.1	42.3	50.7	51.1
Protein	53.5	53.5	42.8	50.9	51.5
Ligands/ions^d^	34.2 (29.1)	34.0 (31.7)	28.4 (27.9)	53.8 (37.6)	44.4 (37.7)
Water	35.3	40	33.5	32.1	42.0
rmsd from ideal values:					
Bond lengths (Å)	0.006	0.006	0.006	0.006	0.006
Bond angles (°)	0.96	0.94	0.95	1.04	0.98

^a^Epac2, *mus musculus*, amino acids 306–993, data collection statistic for each complex correspond to a dataset derived from a single crystal

^b^Values in parenthesis correspond to highest resolution shell.

^c^For the calculation of the Free-R factor the same reflections were omitted as in the dataset of the first solved structure of Epac2^Δ305^•SpcAMPS•Rap (pdb-entry 3CF6).

^d^Values in parenthesis correspond to the cyclic nucleotide in the CNB domain.

Compared to Epac2^K405Q^•cAMP•Rap the 2′-O-Me group of D-007 pushes away Gln^405^ in Epac2^K405Q^•D-007•Rap (Fig. 3F). In consequence, Gln^405^ forces the hinge into a different and apparently more favorable conformation, in which Glu^443^ forms hydrogen bonds with Gln^405^ and Tyr^480^. Gln^405^ does not form a hydrogen bond with the 2′-O-Me group. The loss of the hydrogen bond explains the affinity reducing effect of the 2′-O-Me-group (compare Z-004 and cAMP). The aromatic ring of the 8-pCPT group is kinked perpendicular to the base and shields the binding pocket against the solvent. The transition of the aromatic ring into the hydrophobic protein environment upon binding favors the interaction, which explains the gain in affinity attributed to the 8-pCPT group (compare L-026 with cAMP).

The mechanism of efficient activation of Epac1 by D-007 is thus dependent on the unique Gln^270^ in Epac1. As this residue is not conserved in Epac2, D-007 is a poor activator of Epac2. This indicates that the differences between Epac1 and Epac2 are sufficient to provide a window for Epac2 selective activation.

### Design of Efficient Epac2 Agonists

**Figure 4:**
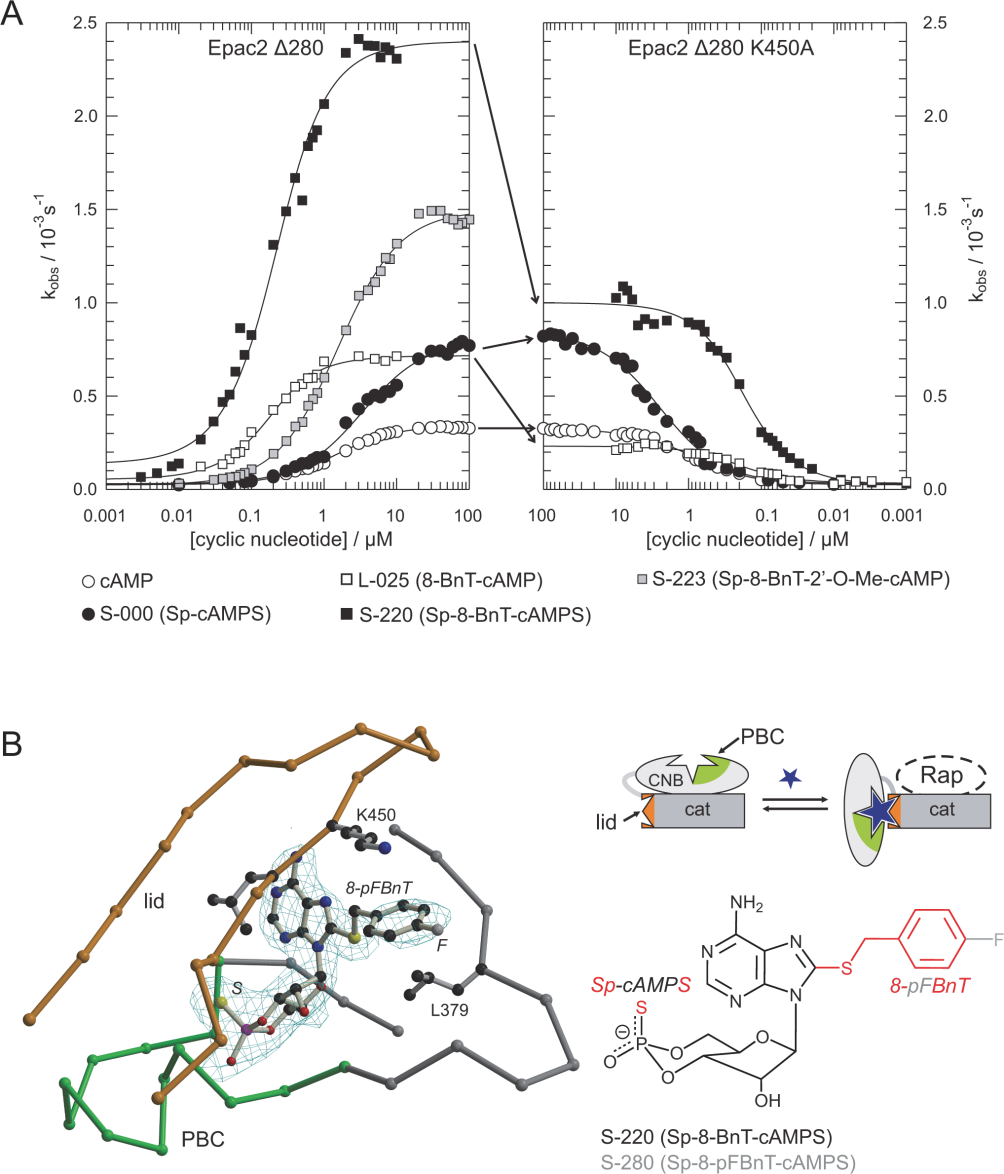
Design of an efficient Epac2 agonist. (A) The maximal activity towards Epac2^Δ280^ is increased in phosphorothioates that contain the sulphur atom in the axial position. The affinity and maximal activity is further increased by the introduction of a BnT-group at the 8-positon. The effect of the BnT-group is selectively eliminated in Epac2^Δ280,K450A^. Plot data can be found in S1 Data. (B) The structure of Epac2•S-280•Rap with *fofc* density calculated in the absence of S-280. The pFBnT-group (the only difference to the BnT group and therefore to S-220 is the fluorine atom (*F*) in para-position) undergoes hydrophobic stacking interaction with Lys^450^ in the lid (orange). *S* marks the sulphur atom of the phosphorothioate.

To identify the properties of cAMP-analogues that efficiently activate Epac2, the chemical space of substitutions was systematically tested. In total, approximately 100 cAMP analogues, half of which were newly synthesized, were characterized in an iterative design process by determining their AC_50_ and their relative k_max_ (Table 1). First, cAMP-analogues modified at a single position were tested. The N-series of analogues in which the N^6^-position is modified shows strongly decreased maximal activities. This characteristic is in agreement with the crucial interactions of the natural NH_2_-group at this position of cAMP in stabilizing the active conformation of Epac [11]. In contrast to Epac1, 2′-modifications, which were tested in the Z-series, do not improve k_max_ values for Epac2, but result in a reduction of affinity by one or two orders of magnitude. The stereo-specific substitution of the axial oxygen in the cyclic phosphate by sulphur (Sp-cAMPS, S-000) results in a relative k_max_ of 2.5 without influencing the affinity (Fig. 4A left panel; Table 1). Twenty-eight different modifications at the 8-position were tested in the L-series. Several modifications like *cyclo*hexylamino group (L-013) reduce the maximal activity. On the other hand, many modifications enhance the maximal activities, whereby relative k_max_ values between 1.5 and 5.1 were obtained. The improved maximal activity is accompanied either by a loss or a gain in affinity.

**Figure 5:**
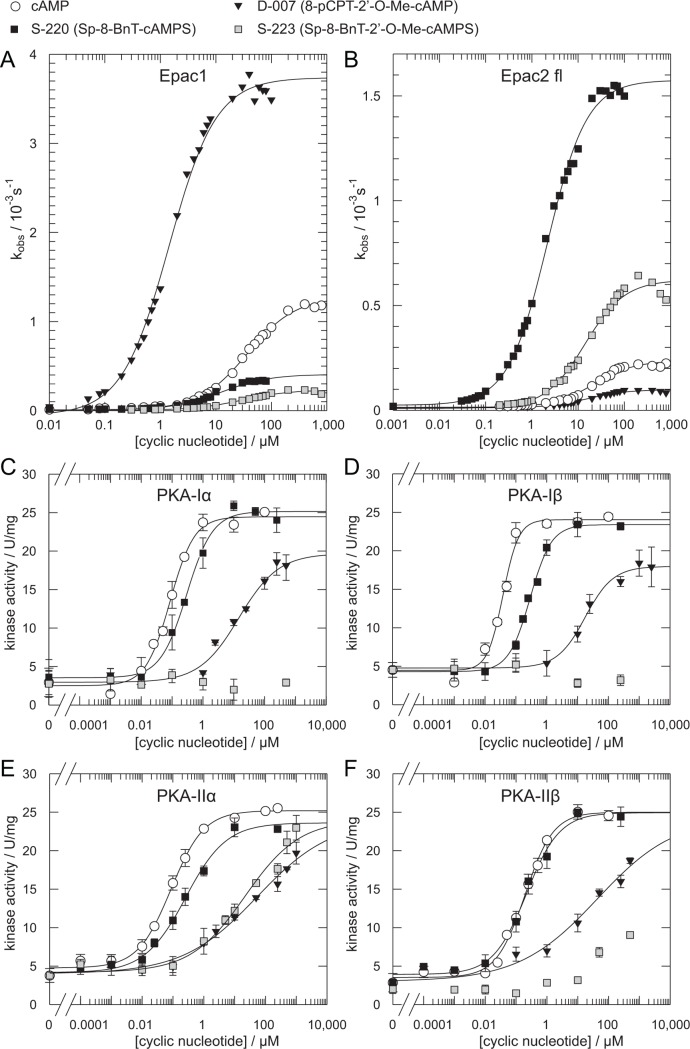
Selective activation of Epac proteins *in vitro*. (A, B) Comparison of cyclic nucleotide-mediated activation of Epac1 and Epac2^fl^. Plot data can be found in S1 Data. (C– F) Cyclic nucleotide mediated activation of PKA-Iα, PKA-Iβ, PKA-IIα, and PKA-IIβ. Each data point represents the mean ± standard deviation (SD) of at least two measurements. Plot data can be found in S1 Data.

Since modifications of the phosphate system and the 8-position show favorable properties, 8-modified versions of S-000 were synthesized to form the S-series. The focus was on 8-modifications, which in addition to an improved maximal activity had shown a gain in affinity in the L-series. Overall the results show that the effects of both modifications on the maximal activity is “additive” resulting in double modified analogues with increased k_max_ values of up to 8. In addition, gains in affinity mediated by the 8-modifications are maintained. This finding is illustrated in the left panel of Fig. 4A for S-220, in which a benzylthio group (BnT) was introduced as 8-modification in S-000. S-220 is one of the most potent Epac2 activators, which activates Epac2 with an AC_50_ of 0.1 μM and a relative k_max_ of 7.7 (1.8 μM and 1 for cAMP). S-220 was selected for a more detailed analysis as it functions as an efficient activator of Epac2 and its biophysical characterization indicated it as a promising candidate to activate Epac2 selectively over Epac1 (Fig. 5A; Table 1). Additional substitutions at the benzyl ring (S-230, S-240, S-250, S-260, S-270, S-280, S-290, and S-300) do not result in improvements compared to S-220 (Table 1). Similarly, a benzylthio group was the most favorable choice if compared to benzylamino, benzyloxy, and benzylseleno groups (compare L-025 with L-015, L-023 and L-028) (Table 1).

To understand the molecular basis of efficient Epac-2 activation the crystal structures of the complexes containing S-220 and S-280 were solved (Fig. 4B; Table 2). The structure with S-280, which differs from S-220 by only one fluorine atom as a substituent at the para position in the benzyl ring, is shown in Fig. 4B, because it was solved at a higher resolution and does not differ from the Epac2 structure with S-220. The backbone conformation of these structures did not differ from that containing cAMP. Unlike in Epac2^K405Q^ (the model of Epac1) efficient activation is thus not caused by differences in the hinge.

Also, the surroundings of the thiophosphate did not differ. The interaction of the phosphate system with the protein initiates the conformational changes leading to the movement of the CNB-domain required for activation [7,10]. Apparently, the physical properties of the sulphur in the axial position are favored over the oxygen in the active conformation of Epac2. The inability of Rp-cAMPS (R-000) (sulphur in the equatorial position of the cyclic phosphate) to activate Epac emphasizes once more the sensitivity of the cyclic phosphate to perturbations [13].

The aromatic ring of the BnT/pFBnT-group of S-220/S-280 is sandwiched between Leu^379^ and Lys^450^. Leu^379^ is part of the core CNB domain, and Lys^450^ is part of the lid. The BnT-group “glues” the lid to the core and thereby stabilizes the active conformation. Indeed, the contribution of the BnT-group to efficient activation is lost in Epac2^Δ280,K450A^ (Fig. 4A). The k_max_-value of L-025 (8-BnT-cAMP) is reduced to that of cAMP and the k_max_-value of S-220 (Sp-8-BnT-cAMPS) is reduced to that of S-000 (Sp-cAMPS) (Fig. 4A). Therefore, the effects of the thiophosphate and the 8-BnT-modification are of different origin and can be separated.

For a final validation, also the activation curves of full length Epac2 (Epac2^fl^) were recorded in direct comparison to Epac1 (Fig. 5A). S-220 is an efficient activator of Epac2^fl^ but a poor activator of Epac1 (Fig. 5A). The concentration of an analogue required to reach the half-maximal activity of cAMP can be defined as the activation potential (Fig. 1D). This definition reflects the effects on the maximal activity as well as on the affinity. By this definition, S-220 activates Epac2^fl^ 200-times more potently than cAMP, but cannot activate Epac1 to the level of half-maximal cAMP-activity. Conversely, D-007 activates Epac1 100-times more potently than cAMP and reaches half-maximal cAMP activity with Epac2 only under saturating conditions.

Glu^315^ of Epac1 corresponds to Lys^450^ in Epac2. The ability of the 8-BnT-substitution to induce a high maximal activity of Epac2 is lost in Epac2^K450E^, which shows an activation behavior very similar to that of Epac2^K450A^ (S1 Fig.). Thus, the preference of 8-BnT-substituted analogues for Epac2 at least partially originates from this difference.

### Discrimination against PKA

For biological applications, Epac-selective cAMP analogues should not act on PKA. S-220 activates all four PKA isoforms in a biochemical kinase assay (Fig. 5C–5F). This is in agreement with the earlier findings that PKA is activated by several cAMP analogues with bulky modifications at the 8-position [38–40] and tolerates axial phosphorothioates [40,41]. However, PKA-Iα, PKA-Iβ, and PKA-IIα are activated with a K_act_ three to eight times higher than that of cAMP, whereas PKA-IIβ is activated by S-220 and cAMP with comparable affinities (Fig. 5C–5F; S2 Table).

Modifications of the 2′-OH group effectively discriminate against PKA [34,35,42]. Therefore, several substitutions of the 2′-OH group, such as hydrogen, chlorine, fluorine, or Me-O, were introduced in 8-substituted Sp-cAMPS analogues. All 2′-substitutions decreased the maximal activity and affinity of Epac2 activation compared to the corresponding un-substituted mother compound (Table 1). S-223 is the most potent 2′-substituted Epac2 activator that efficiently discriminates against Epac1 (Fig. 5A and 5B; Table 1). S-223 still activated Epac2 ten times more potently than cAMP (Fig. 5B). Conversely, the ability of S-223 to activate PKA was drastically reduced (Fig. 5C–5F). PKA-Iα and PKA-Iβ were not activated at concentrations up to 1 mM. Furthermore, the K_act_ of PKA-IIα and PKA-IIβ is reduced approximately 300 and 11,000 times, respectively, compared to cAMP (Fig. 5C–5F; S2 Table). Therefore, S-223 discriminates even more efficiently against PKA than D-007, whose affinity is only reduced 250 to 900 times compared to cAMP (Fig. 5C–5F; S2 Table).

### Selectivity in Cellular Systems

**Figure 6:**
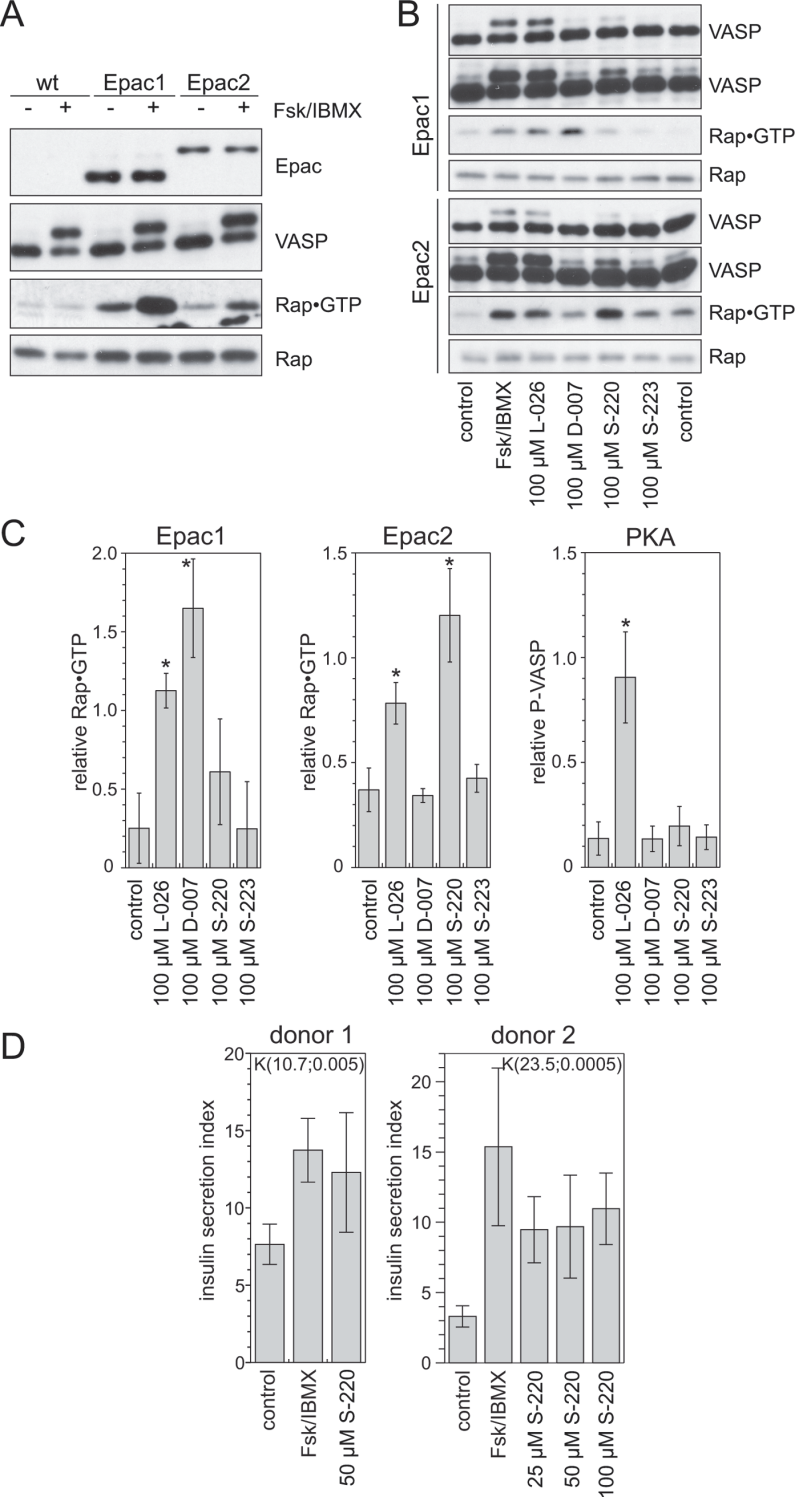
Selective activation of Epac2 results in insulin secretion. (A) Comparison of U2OS cell lines stably expressing Epac1 or Epac2 with parent cells. Cells were mock-stimulated or received 15 μM forskolin and 200 μM IBMX to elevate intracellular cAMP levels. The activation of PKA was monitored by a phosphorylation-induced band shift of VASP. Rap•GTP was precipitated from cell lysates and compared to the total Rap levels. Epac was visualized by an anti-flag antibody. (B) Stimulation of Epac1 or Epac2 cells with reagents as indicated. To visualize the low activity levels of PKA, long exposures of the VASP blots are shown next to the normal exposure time. (C) Quantification of Western blots obtained from experiments as shown in (B). Rap•GTP and P-VASP levels were determined relative to the induction obtained with forskolin and IBMX. As the response of PKA was indistinguishable in the Epac1 and Epac2 cell lines, P-VASP levels of both cell lines were averaged. Values for L-026 and S-223 are based on two and for D-007 and S-220 on four independent experiments. For statistical analysis data were compared to non-stimulated cells in a two-tailed and unpaired Student’s *t* test; *p < 0.01. Bar-graph data can be found in S1 Data. (D) Primary human islets isolated from donor pancreas were stimulated with reagents as indicated, and insulin secretion was determined as the ratio of insulin level before and after stimulation (insulin secretion index). A Kruskal-Wallis test was performed for statistical analysis, which is depicted as K(χ^2^;*p*-value) in the graphs. Bar-graph data can be found in S1 Data.

A model cell-system was generated to confirm the *in vitro* selectivities under more physiological conditions (Fig. 6A). U2OS cells do not express Epac1 or Epac2. Therefore increased cAMP levels induced PKA but not Epac signaling. Increased cAMP levels in U2OS cell lines with a stable over-expression of Epac1 or Epac2 resulted in increased Rap•GTP levels next to enhanced PKA signaling. The phosphorylation of vasodilator-stimulated phosphoprotein (VASP) was monitored as a measure of PKA activity by a band shift.

The effect of increased cAMP levels upon the stimulation of cells could be mimicked by the nonselective analogue L-026 (Fig. 6B and 6C). D-007 strongly activated Epac1 but had only minor effects on Epac2 and PKA. S-220 efficiently activated Epac2 with only minor effects on Epac1. Interestingly, S-220-induced PKA activation was very low in the cellular model system. In biochemical assays with recombinant proteins S-220 activated PKA with a slightly lower affinity than cAMP, whereas L-026 activated PKA [40] and Epac (Table 1) at lower concentrations than cAMP. Likely, the ultimate cellular concentrations of S-220 were not sufficient to efficiently activate PKA. Unfortunately, S-223 induced neither PKA nor Epac signaling. Its potency is likely further limited by inefficient cellular uptake.

Epac is known to occur in signaling complexes with PKA-anchoring proteins [43]. To investigate a putative contribution of PKA signaling to the formation of Rap•GTP, cells were stimulated after pretreatment with a PKA inhibitor. Inhibition of PKA abolished the phosphorylation of VASP, when cAMP levels were increased by the application of forskolin and 3-isobutyl-1-methylxanthine (IBMX), but had no effect on Rap•GTP levels. Similarly, inhibition of PKA had no effect of S-220 or D-007 induced Rap activation (S2 Fig.).

### S-220 Augments Insulin Secretion

To test the potential of S-220 to augment glucose-induced insulin secretion primary islets were isolated from human pancreases. The islets were stimulated either with glucose as control or with glucose and Forskolin/IBMX or S-220 (Fig. 6D). Forskolin/IBMX was used as a cAMP inducing agent and represents the maximal possible effect resulting from activation of PKA and Epac2. At concentrations of 25 to 100 μM, S-220 potentiates glucose-induced insulin secretion with similar efficiency (Fig. 6D), suggesting a strong contribution of Epac2.

## Discussion

Selective targeting of highly related proteins is a common challenge in drug design. Here we have targeted the CNB domain of Epac2 in an iterative design process. A comprehensive activity profile was generated by determining the affinity and the relative maximal activity of about 100 cAMP analogues (Table 1). This approach led to the identification of positions in cAMP on which modifications are tolerated by Epac proteins. For example, Epac2 does not tolerate any modification of the amino-group at the 6-position, whereas a wide variety of substituents are tolerated at the 8-position. Different substituents at the 8-position could be used to modulate the affinity and the maximal activity, whereby some modifications increased the maximal activity by a factor of 5. An independent improvement in maximal activity was obtained by introducing a sulphur atom into the axial position (Sp-isomer) of the cyclic phosphate. Interestingly, the effects of the thiophosphate and 8-substitutions are “additive” (Fig. 4A; Table 1). The crystal structure of S-220 in complex with Epac2 shows that in this case the benzyl ring of the 8-substituent stabilizes the CNB domain in the active conformation by hydrophobic interactions. Upon disruption of this interaction by site directed mutagenesis the effect of the 8-subsitution was selectively eliminated.

A similar interaction of an 8-substituent is impossible in Epac1 due to a single amino acid difference. In fact neither 8-substituted cAMP analogues nor Sp-cAMPS analogues are able to increase the maximal activity of Epac1. An increase could only be obtained by modifications of the 2′-position as for example by the 2′-O-Me group in D-007. On the other hand, it was not possible to obtain beneficial effects with 2′-substituted analogues for Epac2. Instead 2′-substitutions decreased the maximal activity of Epac2. Again it was possible to attribute these differences to a single amino acid difference between Epac1 and Epac2. Thus, even though highly related, Epac isozymes show distinct activity profiles, which originate from subtle differences in the cAMP binding site. In consequence of these differences fundamentally different effects are responsible to stabilize the CNB domain in the active conformation of Epac1 and Epac2.

For structural studies we used Epac2^K405Q^ as a model of Epac1. Epac2^K405Q^ mimics the response of Epac1 towards 2′-substituted cAMP analogues and is efficiently activated by D-007, while Epac2 is not (Fig. 1C). The advantage of this approach is, that any differences in the structures if compared to Epac2 must originate exclusively from the single amino acid point mutation. The 2′-O-Me-group of D-007 induces via the glutamine a different conformation in the backbone of the hinge. Thus, upon the transition from the inactive to the active state the conformation of the hinge is transformed into different, more or less favored, conformations depending on the nature of the cyclic nucleotide.

In depth characterization classified D-007 as an efficient activator of Epac1 and S-220 as an efficient activator of Epac2 (Fig. 4A and 4B). Actually, the natural agonist cAMP appears to be a rather poor activator of Epac compared to D-007 or S-220. Assuming that the most efficient activators shift the equilibrium between the inactive and the active conformation (almost) fully to the active side, about 65% and 85% of cAMP bound Epac1 and Epac2, respectively, would still be in an inactive conformation (Fig. 1D). This property of Epac eases the design of analogues, as a better activation potential can be gained by improving affinity and maximal activity. During evolution the key requirement for Epac might have been to be cAMP responsive rather than making optimal use of the catalytic potential. Alternatively, any factor that binds specifically to the active conformation of Epac would shift the equilibrium to the active side. Although the existence of such a factor is speculative, it could add an extra level of regulation by exhausting the full catalytic potential.

S-220 activates all isoforms of PKA in biophysical assays, though less efficiently than cAMP (Fig. 5C–5F). Discrimination of S-220 against PKA and Epac1 is thus not absolute. However, the capability of S-223, a variant of S-220, to activate PKA is basically absent in biophysical assays. But it must be noted that S-223 is over 20 times less potent in activating Epac2 and it turned out that S-223 is unable to induce Epac2 activation in cell culture. Interestingly, the selectivity window provided by S-220 is sufficient to cause selective activation of Epac2 *in vivo*. Similarly, D-007 seems to act as an Epac1 selective agonist, despite its low potential to activate Epac2 in biophysical assays (Fig. 5). The bioavailability of the analogues limits the maximal concentration that is reached in the cell. Thus the optimal compromise between bioavailability on the one site and biophysically defined selectivity and activation potential on the other site needs to be identified and validated as demonstrated in the case of S-220 and S-223. To avoid putative cross-reactivity it is in any case advisable to use the analogues at the lowest possible concentration.

D-007 was originally introduced as an Epac selective cAMP-analogue due to its poor activation potential for PKA [34,35]. However, direct data were only obtained with Epac1 [13,34] and a comprehensive biophysical analysis had introduced the concept of efficient activation based on the characterization of Epac1 [12,13]. In fact, D-007 is a poor activator of Epac2 (Figs. 1B and 4B). D-007 induces no or marginal Epac2 activation in our model system of Epac cell lines (Fig. 6B and 6C). Interestingly, several studies have used D-007 to investigate Epac2 mediated biological effects (for example [24,25,44,45]). In these studies, D-007 was frequently applied at rather high concentrations as an acetoxymethyl ester. This ester functions as a pro-drug with improved membrane permeability and releases the active mother compound in the cell. It was shown to act at 100- to 1,000-fold lower concentration in tissue culture if compared with the direct application of D-007 [46]. The acetoxymethyl ester of D-007 might therefore be capable of causing sufficient Epac2 activation if applied at high concentrations.

The combination of cAMP analogues with recently developed Epac inhibitors [47] will ease distinguishing Epac1, Epac2, and PKA-mediated effects. D-007, S-220, and N-002 act as selective agonists of Epac1, Epac2, and PKA, respectively, and can be used in direct comparison in titration experiments. N-002 was previously shown to activate PKA but not Epac1 [35], and this study demonstrates also its inability to activate Epac2 (Table 1). Inhibitors targeting the kinase domain of PKA allow selective inhibition of PKA-mediated signaling [48–51]. ESI-05 and its derivative HJC0350 are selective inhibitors of Epac2 [52–54] and CE3F4 inhibits preferentially Epac1 over Epac2 [55,56].

S-220 augments glucose induced insulin secretion from primary human islets. Selective activation of Epac2 under physiological conditions by cAMP analogues is thus feasible. S-220 showed a similar potential as agents that induce cAMP production (Fig. 6). This argues for a major role of Epac2 in mediating the effects of GIP and GLP-1 on direct insulin secretion. S-220 is therefore expected to become a valuable tool in analyzing the underlying signaling pathways in more detail. The pharmacological properties of cAMP analogues are not optimal, in particular as their membrane permeability is limited by the negative charge of the cyclic phosphate. It is, however, possible to convert cAMP analogues into pro-drugs, in which the negative charge is masked. This concept was proven by the previously mentioned acetoxymethyl ester of D-007, while Adefovir dipivoxil is an example of a related pro-drug version of a 5′-AMP analogue for the treatment of hepatitis B.

In summary, we have selectively targeted the highly related CNB domains of Epac1 and Epac2 by cAMP analogues. Several analogues are capable of activating Epac2 up to 200-fold higher potency than cAMP. The structural basis of this efficient activation and of selectivity was identified. Our data indicate that *in vivo* D-007 and S-220 are selective agonists of Epac1 and Epac2, respectively. S-220 potentiates insulin secretion in primary human islets, confirming a major role of Epac2 in this process. The results obtained for S-220 indicates that selective pharmacological targeting of Epac2 is possible.

## Material and Methods

### Ethics Statement

Human cadaveric donor pancreases were procured via a multi-organ donation program. Islets were isolated at the Leiden University Medical Center and were used in this study if they could not be used for clinical transplantation, according to national laws, and if research consent was present.

### Epac Activation Assay

The following constructs were used: Epac1, *homo sapiens*, amino acids 150–881, referred to as Epac1; Epac2, *mus musculus*, amino acids 1–993 and amino acids 281–993, referred to as Epac2^fl^ and Epac2^Δ280^, respectively. Murine Epac2 was used as all previous structural studies were performed with it [10,11,57]. Murine Epac2 is more than 97% identical to human Epac2, and the CNB site is 100% identical.

The activity of Epac was determined by a fluorescence assay [12]. In brief, the substrate protein Rap1B was loaded with the fluorescent GDP analogue 2′-/3′-O-(N-methylanthraniloyl)-guanosine diphosphate (mGDP). The fluorescence intensity of Rap1B•mGDP is approximately twice that of free mGDP, and thus, nucleotide exchange can be observed as a decay in fluorescence upon the addition of excess unlabeled GDP. The decay is single exponential, and the observed rate constant was plotted against the concentration of cyclic nucleotide (Figs. 1C, 4A, 5A, and 5B).

### PKA Activity Assay

The recombinant human PKA catalytic subunit (Cα1) and the four human PKA regulatory subunits (RIα, RIβ, RIIα, RIIβ) were expressed, purified, and characterized as described [58]. The PKA activity was assayed using a coupled spectrophotometric assay [59] with 260 μM Kemptide (LRRASLG; GeneCust) as a substrate and cyclic nucleotides in a range from 100 pM to 1 mM. PKA holoenzymes were formed by mixing the R- and C-subunit at a molar ratio of 1.2:1 and extensive dialysis against 20 mM MOPS (pH 7.0), 150 mM NaCl, 2.5 mM β-mercaptoethanol, supplemented with 1 mM ATP and 10 mM MgCl_2_ for PKA-I at 4°C overnight. PKA holoenzymes were used at about 20 nM for the activation assay. The apparent activation constants (K_act_) were determined by fitting the concentration-dependent activity to a sigmoid dose-response model.

### Cellular Epac and PKA Activity Assay

Rap•GTP levels were determined by precipitating GTP-bound Rap specifically from cell lysates and subsequent western Blotting with an α-Rap antibody (Santa Cruz Biotechnology) [46]. To generate stable cell lines, U2OS cells were transfected with pBabe-Flag-Epac1 (Epac1, *homo sapiens*, amino acids 1–881) or pBabe-Flag-Epac2 (Epac2, *mus musculus*, amino acids 1–993), selected for Epac expression and maintained under selection with 2 mg/l puromycin. The cells were cultured according to standard protocols. The activity of PKA was determined by western blotting with a monoclonal α-VASP antibody (BD Transduction Laboratories). Blots were developed by enhanced chemiluminescence (ECL) and the use of X-ray films; for quantification films were scanned. The intensities of the Rap•GTP bands and the upper band of VASP (P-VASP) were determined for each condition in ImageJ and normalized to the stimulation with forskolin and IBMX for each blot.

### Crystallography

Epac2 proteins (Epac2, *mus musculus*, amino acids 306–993) were purified and crystallized as described [10].

### Insulin Secretion Assay

Islets were isolated from two human donor pancreases [60]. Intact islets (*n* = 20 per well) were seeded on ultra-low attachment 96-well plate. After 3 days of culture, the pancreatic islets were washed two times with 115 mM NaCl, 5 mM KCl, 24 mM NaHCO_3_, 2.2 mM CaCl_2_, 1 mM MgCl_2_, 20 mM HEPES, and 0.2% human serum albumin (incubation buffer) and primed for 1.5 h at 37°C in incubation buffer supplemented with 2 mM glucose (low glucose). Subsequently, the cells were incubated in fresh incubation buffer supplemented with 2 mM glucose for 1 h to measure the basal insulin secretion levels. The cells were then incubated for 1 h in incubation buffer supplemented with 16.7 mM glucose (high glucose) in the presence or absence of cAMP analogues. The cell supernatants were collected immediately after incubation with low and high glucose. The insulin concentrations were measured by ELISA (Insulin ELISA Kit, Mercodia). The insulin secretion was expressed as the ratio of insulin concentration at high and low glucose concentrations for each well (insulin secretion index).

### Synthesis of cAMP Analogues

All reagents were of analytical grade or the best grade available from commercial suppliers. DMSO was stored over activated molecular sieves (3 Å) for at least two weeks before use. The UV spectra were recorded with a Helios β spectrometer (Spectronic Unicam) in aqueous phosphate buffer (pH 7.0). The mass spectra were obtained with an Esquire LC 6000 spectrometer (Bruker Daltonik) in the ESI-MS mode with 50% isopropanol/49.9% water/0.1% formic acid as matrix.

If not stated otherwise, all chromatographic operations were performed at ambient temperature. The analytical HPLC-system consisted either of a L-6200 pump, a L-4000 variable wavelength UV/Vis detector and a D-2500 GPC integrator (all Merck-Hitachi) or a LaChrom Elite instrument with a L-2130 pump, a L-2420 variable wavelength UV/Vis detector, a L-2350 column oven (set at 30°C), and EZChrom software version 3.3.1 SP1 (all VWR-Hitachi). The stationary phase was Kromasil (AkzoNobel) C 8–100, 10 μm, or YMC ODS-A 12 nm, S-11 μm (YMC), both in 250 × 4.6 mm stainless steel columns.

Preparative MPLC was accomplished with a C-605 pump (Büchi), a preparative K 2001 UV-detector (Knauer), and a L200E analog recorder (Linseis). Merck LiChroprep RP-18 6 nm, 15–25 μm (Merck-Hitachi) in a 410 × 50 mm glass column (Kronlab) was used to isolate and desalt the nucleotides. If necessary, cation exchange was carried out with Toyopearl SP-650M, 65 μm, sodium form (Tosoh Bioscience), in a 125 × 35 mm or a 250 × 50 mm glass column (Kronlab).

Preparative HPLC was performed with a LC-8A pump (Shimadzu), a preparative K 2001 UV-detector (Knauer), and a L200E analogue recorder (Linseis). YMC ODS-A 12 nm, S-11 μm (YMC) in a 250 × 20 mm or a 250 × 16 mm stainless steel column was used for purification and desalting.

Purification columns (MPLC and HPLC) were equilibrated with either 100 mM NaH_2_PO_4_ or 20 mM triethylammonium formate (TEAF) (pH 7). Subsequently, the raw products were applied and the columns were initially washed with the same buffer, followed by water. Each cyclic nucleotide was eluted with a gradient from 10% water to 20%–50% isopropanol or acetonitrile. Cation exchange to sodium was performed with compounds isolated from the purifications with TEAF buffer during the equilibration phase. The product-containing fractions were collected and evaporated under reduced pressure to obtain the target compound in the sodium form.

The typical yields of isolated cyclic nucleotides were in the range of 20%–85%. The purity of each analogue was at least >99% (by analytic HPLC at λ_max_). The structure of each analogue was confirmed by UV/VIS spectrometry and ESI/MS analysis. The structures of selected analogues were further confirmed by NMR (S1 Text). The nucleotides were quantified and aliquoted using the extinction coefficient at their λ_max_.

The following compounds were provided by BIOLOG LSI: 8-Br-2′-Cl-adenosine, 8-Br-2′-F-adenosine, cAMP, 2′-dcAMP (Z-001), 2′-F-cAMP (Z-002), 2′-NH_2_-cAMP (Z-003), 2′-O-Me-cAMP (Z-004), 8-Cl-cAMP (L-001), 8-Br-cAMP (L-002), 8-NH_2_-cAMP (L-003), 8-N_3_-cAMP (L-004), 8-MeA-cAMP (L-005), 8-AEA-cAMP (L-006), 8-HA-cAMP (L-009), 8-AHA-cAMP (L-010), 8-DMeA-cAMP (L-015), 8-PIP-cAMP (L-018), 8-OH-cAMP (L-021), 8-BnT-cAMP (L-025), 8-pCPT-cAMP (L-026), N^6^-Bn-cAMP (N-001), N^6^-Bnz-cAMP (N-002), N^6^-Phe-cAMP (N-003), 8-Br-2′-O-Me-cAMP (D-005), 8-pCPT-2′-O-Me-cAMP (D-007), 8-pOHPT-2′-O-Me-cAMP (D-008), 8-pMeOPT-2′-O-Me-cAMP (D-009), 8-OH-2′-O-Me-cAMP (D-010), Sp-cAMPS (S-000), Sp-8-Br-cAMPS (S-010), Sp-8-Br-2′-dcAMPS (S-011), Sp-8-Br-2′-O-Me-cAMPS (S-014), Sp-8-Cl-cAMPS (S-020), Sp-8-ADOA-cAMPS (S-060), Sp-8-PIP-cAMPS (S-110), Sp-8-OH-cAMPS (S-130), Sp-8-pCPT-cAMPS (S-210), Sp-8-pCPT-2′-O-Me-cAMPS (S-211), Sp-5,6-DCl-cBIMPS (S-400), Rp-cAMPS (R-000).

2′-O-Pr-cAMP (Z-005), 2′-O-Bu-cAMP (Z-006), 8-OHEA-cAMP (L-007), 8-(*S*-2-OHPrA)-cAMP (L-012), 8-PIA-cAMP (L-019), and Sp-8-PIA-cAMPS (S-120) were provided by B. Jastorff (Bioorganic Chemistry Unit, Department of Chemistry, University of Bremen, Bremen, Germany).

2′-O-All-cAMP (Z-007) and 2′-O-Bn-cAMP (Z-008) were prepared from cAMP by alkylation with appropriate alkylhalogenides [61].

8-Br-2′-Cl-cAMP (D-003), 8,2′-DCl-cAMP (D-006), and 8-Br-2′-F-cAMP (D-004) were prepared from 8-Br-2′-Cl-adenosine and 8-Br-2′-F-adenosine by a two-step one-pot reaction scheme with 2 equivalents (eq.) phosphoryl chloride and excess cyclisation solution consisting of 0.1% KOH in acetonitrile/water 60/40 (v:v) at room temperature [62]. 8,2′-DCl-cAMP was obtained as a side product during 8-Br-2′-Cl-cAMP production by bromine to chlorine exchange in position 8 of the adenine moiety. Chlorine was introduced in the initial phosphorylation step as verified by analytical HPLC.

Sp-8-Br-2′-Cl-cAMPS (S-013), Sp-8,2′-DCl-cAMPS (S-021), and Sp-8-Br-2′-F-cAMPS (S-012) were synthesized from Br-2′-Cl-adenosine and 8-Br-2′-F-adenosine by a related two-step one-pot reaction scheme with thiophosphoryl chloride and refluxing cyclisation solution [63]. Sp-8,2′-DCl-cAMPS was pre-formed as a side product during the thiophosphorylation of 8-Br-2′-Cl-adenosine.

8-MeSe-cAMP (L-027), 8-BnSe-cAMP (L-028), 8-BnSe-2′-O-Bn-cAMP (D-012), and 8-BnSe-2′-O-Me-cAMP (D-013) were prepared from corresponding 8-bromo nucleotides by nucleophilic substitution with sodium hydrogen selenide [64] and subsequent alkylation with methyl iodide or benzyl bromide as described [65].

#### Scheme A

8-Amino-substituted analogues were prepared via the nucleophilic substitution of 8-bromo-substituted cAMP or cAMPS analogues as described [66] with some modifications.

Typical reactions were performed with 100–1,000 μmol of 8-bromo-substituted starting nucleotide with 50–200 eq. of amine reagent dissolved in 2–50 ml water with variable amounts of isopropanol depending on the lipophilicity of the particular amine reagent to ensure sufficient solubility. The reaction mixtures were refluxed until the starting material was no longer detectable by HPLC analysis, diluted with water, and extracted three times with dichloromethane. After neutralization with diluted HCl, the aqueous phase was concentrated by rotary evaporation under reduced pressure and purified as described above.

The following analogues were produced by scheme A: 8-AllA-cAMP (L-011), 8-cHA-cAMP (L-013), 8-BnA-cAMP (L-014), 8-DEA-cAMP (L-016), 8-Pyr-cAMP (L-017), 8-Morph-cAMP (L-020), 8-MeA-2′-Cl-cAMP (D-001), Sp-8-MeA-cAMPS (S-030), Sp-8-MeA-2′-dcAMPS (S-031), Sp-8-MeA-2′-F-cAMPS (S-032), Sp-8-MeA-2′-Cl-cAMPS (S-033), Sp-8-MeA-2′-O-Me-cAMPS (S-034), Sp-8-EA-cAMPS (S-040), Sp-8-iPrA-cAMPS (S-050), Sp-8-DMeA-cAMPS (S-070), Sp-8-EMeA-cAMPS (S-080), Sp-8-DEA-cAMPS (S-090), and Sp-8-iPrMeA-cAMPS (S-100).

#### Scheme B

The 8-thio-substituted analogues were generated via the nucleophilic substitution of 8-bromo-substituted nucleotides with aryl- and alkylthiol reagents.

Fifty to 1,000 μmol halogen-containing starting nucleotide, 1.5–3 eq. thiol reagent and 1.2 eq. NaOH or diisopropylethylamine (DIEA) were dissolved in 1–50 ml water with variable amounts of isopropanol to improve the dissolution of reactants. The mixtures were vigorously stirred and heated to 70–100°C until no further reaction progress was detected by analytical HPLC. After dilution with water, extraction three times with dichloromethane and three times with ethyl acetate, the aqueous phase was neutralized with HCl, concentrated and the reaction product was purified as described above.

The following analogues were produced by scheme B: 8-MeT-cAMP (L-024), 8-BnT-2′-F-cAMP (D-011), Sp-8-MeT-cAMPS (S-160), Sp-8-MeT-2′-F-cAMPS (S-170), Sp-8-MeT-2′-O-Me-cAMPS (S-180), Sp-8-PheET-cAMPS (S-190), Sp-8-pOHPT-cAMPS (S-200), Sp-8-pOHPT-2′-F-cAMPS (S-201), Sp-8-pOHPT-2′-O-Me-cAMPS (S-202), Sp-8-BnT-cAMPS (S-220), Sp-8-BnT-2′-dcAMPS (S-221), Sp-8-BnT-2′-F-cAMPS (S-222), Sp-8-BnT-2′-O-Me-cAMPS (S-223), Sp-8-pMeBnT-cAMPS (S-230), Sp-8-tBuBnT-cAMPS (S-240), Sp-8-pMeOBnT-cAMPS (S-250), Sp-8-pCBnT-cAMPS (S-260), Sp-8-oCBnT-cAMPS (S-270), Sp-8-pFBnT-cAMPS (S-280), Sp-8-mNBnT-cAMPS (S-290), Sp-8-mTFMeBnT-cAMPS (S-300). Noteworthy, 8,2′-DMeT-cAMP (D-002) with two thiol-containing substituents is the major product formed in the reaction of 8-Br-2′-Cl-cAMP with methanethiol. In our hands, scheme B is not applicable to the synthesis of the mono methylthio-substituted 8-MeT-2′-Cl-cAMP.

#### Scheme C

8-alkoxy-substituted analogues were prepared via the nucleophilic substitution of 8-bromo-substituted nucleotides with methyl- and benzyl alkoxide.

Routinely, 375 μmol halogen-containing starting nucleotide and 3 eq. methyl alkoxide (30% in methanol) or benzyl alkoxide (1 M in benzyl alcohol) were dissolved in 1 ml DMSO abs. in 3 ml reaction tubes with a screw cap. The mixtures were vigorously shaken and heated to 70°C in a thermomixer until no further reaction progress was detected by analytical HPLC. After dilution with water, extraction three times with dichloromethane and three times with ethyl acetate, the aqueous phase was neutralized with HCl, concentrated and each target compound was purified as described above.

Analogues produced by scheme C: 8-MeO-cAMP (L-022), 8-BnO-cAMP (L-023), Sp-8-MeO-cAMPS (S-140), Sp-8-BnO-cAMPS (S-150).

### NMR Analysis

cAMP analogues were dissolved in D_2_O to a final concentration of 10 mM. Spectra were recorded at 293 K or 298 K (S140) on a 750 MHz Bruker Avance NMR machine equipped with 5mm QXI probe (S1 Text). Reported chemical shifts are calibrated directly (^1^H) or indirectly (^31^P, ^13^C) with respect to DSS. Assignments of S150 and S220 are validated with 2D TOCSY (mixing times of 20 and 100 ms) and [^1^H;^13^C]-HSQC.

## Supporting Information

S1 Data Supporting plot and bar-graph data of Figs. 1C, 4A, 5A–5F, 6C, 6D, and S1. (XLSX)

S1 Fig Epac2^Δ280,K450A^ and Epac2^Δ280,K450E^ respond similarly to cyclic nucleotides. Data for Epac2^Δ280,K450A^ are taken from Fig. 4A. Plot data can be found in S1 Data.
(EPS)

S2 Fig Epac mediated increases in Rap GTP levels are PKA independent. U2OS cell lines stably expressing Epac1 or Epac2 were either mock-treated or treated with 10 μM of the PKA inhibitor H-89. 10 minutes after application of the inhibitor Epac1 and Epac2 cells where stimulated with 100 μM of D-007 and S-220, respectively. In addition cells were mock-stimulation and stimulation 15 μM forskolin and 200 μM IBMX to elevate intracellular cAMP levels. The activation of PKA was monitored by a phosphorylation-induced band shift of VASP. Rap•GTP was precipitated from cell lysates and compared to the total Rap levels.
(EPS)

S1 Table Abbreviations of cAMP analogues. (PDF)

S2 Table Activation constants of PKA. (PDF)

S1 Text NMR spectra of representative cAMP analogues. ^31^P, ^13^C, and ^1^H spectra of D-002, L-027, S-030, S-031, S-140, S-150, S-220, S-222, S-223, and S-280 are shown and tentative assignments are presented.
(PDF)

## Abbreviations

CNB: cyclic nucleotide binding
Epac: exchange protein activated by cAMP
GIP: glucose-dependent insulinotropic peptide/gastric inhibitory peptide
GLP-1: glucagon-like peptide-1
IBMX: 3-isobutyl-1-methylxanthine
mGDP: 2′-/3′-O-(N-methylanthraniloyl)-guanosine diphosphate
pCPT: *para*-chlorophenylthio
PKA: cAMP-dependent protein kinase
VASP: vasodilator-stimulated phosphoprotein
