# Supplementary material for: Structure-Guided Design of Selective Epac1 and Epac2 Agonists
Source: PLoS Biol. 2015 Jan 20;13(1):e1002038. doi: 10.1371/journal.pbio.1002038 (PMC4300089; doi:10.1371/journal.pbio.1002038)
Supplement: S1 Table — (PDF) [file pbio.1002038.s004.pdf]

Table S1. Abbreviations of cAMP analogues

		cAMP	Adenosine-3',5'-cyclic monophosphate
2´-modification	Z-001	2'-dcAMP	2'-Deoxyadenosine-3',5'-cyclic monophosphate
	**Z-002**	**2'-F-cAMP**	2'-Deoxy-2'-fluoroadenosine-3',5'-cyclic monophosphate
	**Z-003**	**2'-NH_2-cAMP**	2'-Deoxy-2'-aminoadenosine-3',5'-cyclic monophosphate
	Z-004	2'-O-Me-cAMP	2'-O-Methyladenosine-3',5'-cyclic monophosphate
	Z-005	2'-O-Pr-cAMP	2'-O-Propyladenosine-3',5'-cyclic monophosphate
	Z-006	2'-O-Bu-cAMP	2'-O-Butyladenosine-3',5'-cyclic monophosphate
	**Z-007**	**2'-O-All-cAMP**	2'-O-Allyladenosine-3',5'-cyclic monophosphate
	**Z-008**	**2'-O-Bn-cAMP**	2'-O-Benzyladenosine-3',5'-cyclic monophosphate
8-modification	L-001	8-Cl-cAMP	8-Chloroadenosine-3',5'-cyclic monophosphate
	L-002	8-Br-cAMP	8-Bromoadenosine-3',5'-cyclic monophosphate
	L-003	8-NH_2-cAMP	8-Aminoadenosine-3',5'-cyclic monophosphate
	L-004	8-N_3-cAMP	8-Azidoadenosine-3',5'-cyclic monophosphate
	L-005	8-MeA-cAMP	8-Methylaminoadenosine-3',5'-cyclic monophosphate
	L-006	8-AEA-cAMP	8-(2-Aminoethylamino)adenosine-3',5'-cyclic monophosphate
	L-007	8-OHEA-cAMP	8-(2-Hydroxyethylamino)adenosine-3',5'-cyclic monophosphate
	L-008	8-OHBuA-cAMP	8-(4-Hydroxybutylamino)adenosine-3',5'-cyclic monophosphate
	L-009	8-HA-cAMP	8-Hexylaminoadenosine-3',5'-cyclic monophosphate
	L-010	8-AHA-cAMP	8-(6-Aminohexylamino)adenosine-3',5'-cyclic monophosphate
	**L-011**	**8-AllA-cAMP**	8-Allylaminoadenosine-3',5'-cyclic monophosphate
	L-012	8-(*S*-2-OHPrA)-cAMP	8-(2-(+)S-Hydroxypropylamino)adenosine-3',5'-cyclic monophosphate
	**L-013**	**8-cHA-cAMP**	8-*cyclo*Hexylaminoadenosine-3',5'-cyclic monophosphate
	L-014	8-BnA-cAMP	8-Benzylaminoadenosine-3',5'-cyclic monophosphate
	L-015	8-DMeA-cAMP	8-Dimethylaminoadenosine-3',5'-cyclic monophosphate
	L-016	8-DEA-cAMP	8-Diethylaminoadenosine-3',5'-cyclic monophosphate
	L-017	8-Pyr-cAMP	8-Pyrrolidinoadenosine-3',5'-cyclic monophosphate
	L-018	8-PIP-cAMP	8-Piperidinoadenosine-3',5'-cyclic monophosphate
	L-019	8-PIA-cAMP	8-Piperazinoadenosine-3',5'-cyclic monophosphate
	**L-020**	**8-Morph-cAMP**	8-Morpholinoadenosine-3',5'-cyclic monophosphate
	L-021	8-OH-cAMP	8-Hydroxyadenosine-3',5'-cyclic monophosphate
	L-022	8-MeO-cAMP	8-Methoxyadenosine-3',5'-cyclic monophosphate
	L-023	8-BnO-cAMP	8-Benzyloxyadenosine-3',5'-cyclic monophosphate
	L-024	8-MeT-cAMP	8-Methylthioadenosine-3',5'-cyclic monophosphate
	L-025	8-BnT-cAMP	8-Benzylthioadenosine-3',5'-cyclic monophosphate
	L-026	8-pCPT-cAMP	8-(4-Chlorophenylthio)adenosine-3',5'-cyclic monophosphate
	**L-027**	**8-MeSe-cAMP**	8-Methylselenoadenosine-3',5'-cyclic monophosphate
	**L-028**	**8-BnSe-cAMP**	8-Benzylselenoadenosine-3',5'-cyclic monophosphate

Table S1. Continuation 1 of 2

	N-001	N^6-Bn-cAMP	N^6-Benzyladenosine-3’,5’-cyclic monophosphate
	N-002	N^6-Bnz-cAMP	N^6-Benzoyladenosine-3’,5’-cyclic monophosphate
	N-003	N^6-Phe-cAMP	N^6-Phenyladenosine-3’,5’-cyclic monophosphate
2´-modification + 8-modification	**D-001**	**8-MeA-2’-Cl-cAMP**	8-Methylamino-2’-deoxy-2’-chloroadenosine-3’,5’-cyclic monophosphate
	**D-002**	**8,2’-DMeT-cAMP**	8,2’-Dimethylthioadenosine-3’,5’-cyclic monophosphate
	**D-003**	**8-Br-2’-Cl-cAMP**	8-Bromo-2’-deoxy-2’-chloroadenosine-3’,5’-cyclic monophosphate
	**D-004**	**8-Br-2’-F-cAMP**	8-Bromo-2’-deoxy-2’-fluoroadenosine-3’,5’-cyclic monophosphate
	D-005	8-Br-2’-O-Me-cAMP	8-Bromo-2’-O-methyladenosine-3’,5’-cyclic monophosphate
	**D-006**	**8,2’-DCl-cAMP**	8,2’-Dichloroadenosine-3’,5’-cyclic monophosphate
	D-007	8-pCPT-2’-O-Me-cAMP	8-(4-Chlorophenylthio)-2’-O-methyladenosine-3’,5’-cyclic monophosphate
	D-008	8-pOHPT-2’-O-Me-cAMP	8-(4-Hydroxyphenylthio)-2’-O-methyladenosine-3’,5’-cyclic monophosphate
	D-009	8-pMeOPT-2’-O-Me-cAMP	8-(4-Methoxyphenylthio)-2’-O-methyladenosine-3’,5’-cyclic monophosphate
	D-010	8-OH-2’-O-Me-cAMP	8-Hydroxy-2’-O-methyladenosine-3’,5’-cyclic monophosphate
	**D-011**	**8-BnT-2’-F-cAMP**	8-Benzylthio-2’-deoxy-2’-fluoroadenosine-3’,5’-cyclic monophosphate
	**D-012**	**8-BnSe-2’-O-Bn-cAMP**	8-Benzylseleno-2’-O-benzyladenosine-3’,5’-cyclic monophosphate
	**D-013**	**8-BnSe-2’-O-Me-cAMP**	8-Benzylseleno-2’-O-methyladenosine-3’,5’-cyclic monophosphate
	S-000	Sp-cAMPS	Adenosine-3’,5’-cyclic monophosphorothioate, Sp- isomer
phosphorothioate with 8- and/or 2´-modification	S-010	Sp-8-Br-cAMPS	8-Bromoadenosine-3’,5’-cyclic monophosphorothioate, Sp- isomer
	S-011	Sp-8-Br-2’dcAMPS	8-Bromo-2’-deoxyadenosine-3’,5’-cyclic monophosphorothioate, Sp- isomer
	**S-012**	**Sp-8-Br-2’-F-cAMPS**	8-Bromo-2’-deoxy-2’-fluoroadenosine-3’,5’-cyclic monophosphorothioate, Sp- isomer
	**S-013**	**Sp-8-Br-2’-Cl-cAMPS**	8-Bromo-2’-deoxy-2’-chloroadenosine-3’,5’-cyclic monophosphorothioate, Sp- isomer
	S-014	Sp-8-Br-2’-O-Me-cAMPS	8-Bromo-2’-O-methyladenosine-3’,5’-cyclic monophosphorothioate, Sp- isomer
	S-020	Sp-8-Cl-cAMPS	8-Chloroadenosine-3’,5’-cyclic monophosphorothioate, Sp- isomer
	**S-021**	**Sp-8,2’-Cl-cAMPS**	8,2’-Dichloroadenosine-3’,5’-cyclic monophosphorothioate, Sp- isomer
	**S-030**	**Sp-8-MeA-cAMPS**	8-Methylaminoadenosine-3’,5’-cyclic monophosphorothioate, Sp- isomer
	**S-031**	**Sp-8-MeA-2’-dcAMPS**	8-Methylamino-2’-deoxyadenosine-3’,5’-cyclic monophosphorothioate, Sp- isomer
	**S-032**	**Sp-8-MeA-2’-F-cAMPS**	8-Methylamino-2’-deoxy-2’-fluoroadenosine-3’,5’-cyclic monophosphorothioate, Sp- isomer
	**S-033**	**Sp-8-MeA-2’-Cl-cAMPS**	8-Methylamino-2’-deoxy-2’-chloroadenosine-3’,5’-cyclic monophosphorothioate, Sp- isomer
	**S-034**	**Sp-8-MeA-2’-O-Me-cAMPS**	8-Methylamino-2’-O-methyladenosine-3’,5’-cyclic monophosphorothioate, Sp- isomer
	**S-040**	**Sp-8-EA-cAMPS**	8-Ethylaminoadenosine-3’,5’-cyclic monophosphorothioate, Sp- isomer
	**S-050**	**Sp-8-iPrA-cAMPS**	8-*iso*Propylaminoadenosine-3’,5’-cyclic monophosphorothioate, Sp- isomer
	S-060	Sp-8-ADOA-cAMPS	8-(8-Amino-3,6-dioxaoctylamino)adenosine-3’,5’-cyclic monophosphorothioate, Sp- isomer
	**S-070**	**Sp-8-DMeA-cAMPS**	8-Dimethylaminoadenosine-3’,5’-cyclic monophosphorothioate, Sp- isomer
	**S-080**	**Sp-8-EMeA-cAMPS**	8-Ethylmethylaminoadenosine-3’,5’-cyclic monophosphorothioate, Sp- isomer
	**S-090**	**Sp-8-DEA-cAMPS**	8-Diethylaminoadenosine-3’,5’-cyclic monophosphorothioate, Sp- isomer
	**S-100**	**Sp-8-iPrMeA-cAMPS**	8-*iso*Propylmethylaminoadenosine-3’,5’-cyclic monophosphorothioate, Sp- isomer
	S-110	Sp-8-PIP-cAMPS	8-Piperidinoadenosine-3’,5’-cyclic monophosphorothioate, Sp- isomer

Table S1. Continuation 2 of 2

phosphorothioate with 8- and/or 2´-modification	S-120	Sp-8-PIA-cAMPS	8-Piperazinoadenosine-3’,5’-cyclic monophosphorothioate, Sp- isomer
	**S-130**	**Sp-8-OH-cAMPS**	8-Hydroxyadenosine-3’,5’-cyclic monophosphorothioate, Sp- isomer
	**S-140**	**Sp-8-MeO-cAMPS**	8-Methoxyadenosine-3’,5’-cyclic monophosphorothioate, Sp- isomer
	**S-150**	**Sp-8-BnO-cAMPS**	8-Benzyloxyadenosine-3’,5’-cyclic monophosphorothioate, Sp- isomer
	**S-160**	**Sp-8-MeT-cAMPS**	8-Methylthioadenosine-3’,5’-cyclic monophosphorothioate, Sp- isomer
	**S-170**	**Sp-8-MeT-2’-F-cAMPS**	8-Methylthio-2’-deoxy-2’-fluoroadenosine-3’,5’-cyclic monophosphorothioate, Sp- isomer
	**S-180**	**Sp-8-MeT-2’-O-Me-cAMPS**	8-Methylthio-2’-O-methyladenosine-3’,5’-cyclic monophosphorothioate, Sp- isomer
	**S-190**	**Sp-8-PheET-cAMPS**	8-Phenylethylthioadenosine-3’,5’-cyclic monophosphorothioate, Sp- isomer
	**S-200**	**Sp-8-pOHPT-cAMPS**	8-(4-Hydroxyphenylthio)adenosine-3’,5’-cyclic monophosphorothioate, Sp- isomer
	**S-201**	**Sp-8-pOHPT-2’-F-cAMPS**	8-(4-Hydroxyphenylthio)-2’-deoxy-2’-fluoroadenosine-3’,5’-cyclic monophosphorothioate, Sp- isomer
	**S-202**	**Sp-8-pOHPT-2’-O-Me-cAMPS**	8-(4-Hydroxyphenylthio)-2’-O-methyladenosine-3’,5’-cyclic monophosphorothioate, Sp- isomer
	S-210	Sp-8-pCPT-cAMPS	8-(4-Chlorophenylthio)adenosine-3’,5’-cyclic monophosphorothioate, Sp- isomer
	S-211	Sp-8-pCPT-2’-O-Me-cAMPS	8-(4-Chlorophenylthio)-2’-O-methyladenosine-3’,5’-cyclic monophosphorothioate, Sp- isomer
	**S-220**	**Sp-8-BnT-cAMPS**	8-Benzylthioadenosine-3’,5’-cyclic monophosphorothioate, Sp- isomer
	**S-221**	**Sp-8-BnT-2’-dcAMPS**	8-Benzylthio-2’-deoxyadenosine-3’,5’-cyclic monophosphorothioate, Sp- isomer
	**S-222**	**Sp-8-BnT-2’-F-cAMPS**	8-Benzylthio-2’-deoxy-2’-fluoroadenosine-3’,5’-cyclic monophosphorothioate, Sp- isomer
	**S-223**	**Sp-8-BnT-2’-O-Me-cAMPS**	8-Benzylthio-2’-O-methyladenosine-3’,5’-cyclic monophosphorothioate, Sp- isomer
	**S-230**	**Sp-8-pMeBnT-cAMPS**	8-(4-Methylbenzylthio)adenosine-3’,5’-cyclic monophosphorothioate, Sp- isomer
	**S-240**	**Sp-8-ptBuBnT-cAMPS**	8-(4-*tert*Butylbenzylthio)adenosine-3’,5’-cyclic monophosphorothioate, Sp- isomer
	**S-250**	**Sp-8-pMeOBnT-cAMPS**	8-(4-Methoxybenzylthio)adenosine-3’,5’-cyclic monophosphorothioate, Sp- isomer
	**S-260**	**Sp-8-pCBnT-cAMPS**	8-(4-Chlorobenzylthio)adenosine-3’,5’-cyclic monophosphorothioate, Sp- isomer
	**S-270**	**Sp-8-oCBnT-cAMPS**	8-(2-Chlorobenzylthio)adenosine-3’,5’-cyclic monophosphorothioate, Sp- isomer
	**S-280**	**Sp-8-pFBnT-cAMPS**	8-(4-Fluorobenzylthio)adenosine-3’,5’-cyclic monophosphorothioate, Sp- isomer
	**S-290**	**Sp-8-mNBnT-cAMPS**	8-(3-Nitrobenzylthio)adenosine-3’,5’-cyclic monophosphorothioate, Sp- isomer
	**S-300**	**Sp-8-mTFMeBnT-cAMPS**	8-(3-Trifluoromethylbenzylthio)adenosine-3’,5’-cyclic monophosphorothioate, Sp- isomer
	S-400	Sp-5,6-DCl-cBIMPS	5,6-Dichlorobenzimidazole riboside-3’,5’ cyclic monophosphorothioate, Sp- isomer
	R-000	Rp-cAMPS	Adenosine-3’,5’-cyclic monophosphorothioate, Rp- isomer

Analogues printed in bold were synthesised for the first time, to our knowledge, during this study. For a visualisation of the chemical structures see Figure 2.
