# Supplementary material for: Structure-Guided Design of Selective Epac1 and Epac2 Agonists
Source: PLoS Biol. 2015 Jan 20;13(1):e1002038. doi: 10.1371/journal.pbio.1002038 (PMC4300089; doi:10.1371/journal.pbio.1002038)
Supplement: S2 Table — (PDF) [file pbio.1002038.s005.pdf]

Table S2. Activation constants of PKA

**cyclic nucleotide**	**K_{act} / µM**			
	PKA-Iα	PKA-Iβ	PKA-IIα	PKA-IIβ
cAMP	0.085	0.038	0.080	0.19
D-007	14	18	>70	50
S-220	0.29	0.29	0.27	0.21
S-223	>1,000	>1,000	>25	>1,000
Literature:				
cAMP	0.08 - 0.2 [1-10]	0.02 – 0.04 [6,8,11]	0.09 – 0.19 [6-8,10,12]	0.16 – 0.9 [6-9, 12]
D-007	12 [13]		14 [13]	

[1] Herberg, F. W., Dostmann, W. R., Zorn, M., Davis, S. J. & Taylor, S. S. Crosstalk between domains in the regulatory subunit of cAMP-dependent protein kinase: influence of amino terminus on cAMP binding and holoenzyme formation. *Biochemistry* **33**, 7485-7494 (1994).

[2] Herberg, F. W., Taylor, S. S. & Dostmann, W. R. Active site mutations define the pathway for the cooperative activation of cAMP-dependent protein kinase. *Biochemistry* **35**, 2934-2942 (1996).

[3] Herberg, F. W., Doyle, M. L., Cox, S. & Taylor, S. S. Dissection of the nucleotide and metal-phosphate binding sites in cAMP-dependent protein kinase. *Biochemistry* **38**, 6352-6360 (1999).

[4] Gibson, R. M., Ji-Buechler, Y. & Taylor, S. S. Interaction of the regulatory and catalytic subunits of cAMP-dependent protein kinase. Electrostatic sites on the type Ialpha regulatory subunit. *J. Biol. Chem.* **272**, 16343-16350 (1997).

[5] Ringheim, G. E. & Taylor, S. S. Dissecting the domain structure of the regulatory subunit of cAMP-dependent protein kinase I and elucidating the role of MgATP. *J. Biol. Chem.* **265**, 4800-4808 (1990).

[6] Diskar, M. et al. Regulation of cAMP-dependent protein kinases: the human protein kinase X (PrKX) reveals the role of the catalytic subunit alphaH-alphaI loop. *J. Biol. Chem.* **285**, 35910-35918 (2010).

[7] Moll, D. et al. Biochemical characterization and cellular imaging of a novel, membrane permeable fluorescent cAMP analog. *BMC. Biochem.* **9**, 18 (2008).

[8] Chepurny, O. G. et al. Stimulation of Proglucagon Gene Expression by Human GPR119 in Enteroendocrine L-cell Line GLUTag. *Mol. Endocrinol.* **27**, 1267-1282 (2013).

[9] Vetter, M. M. et al. The testis-specific Calpha2 subunit of PKA is kinetically indistinguishable from the common Calpha1 subunit of PKA. *BMC. Biochem.* **12**, 40 (2011)

[10] Diskar, M., Zenn, H.M., Kaupisch, A., Prinz, A. & Herberg, F.W. Molecular basis for isoform-specific autoregulation of protein kinase A. *Cell. Signal*. **19**, 2024-2034 (2007).

[11] Cadd, G. G., Uhler, M. D. & McKnight, G. S. Holoenzymes of cAMP-dependent protein kinase containing the neural form of type I regulatory subunit have an increased sensitivity to cyclic nucleotides. *J. Biol. Chem.* **265**, 19502-19506 (1990).

[12] Zhang, P. et al. Structure and allostery of the PKA RIIbeta tetrameric holoenzyme. *Science* **335**, 712-716 (2012).

[13] Christensen, A. E. et al. cAMP analog mapping of Epac1 and cAMP kinase. Discriminating analogs demonstrate that Epac and cAMP kinase act synergistically to promote PC-12 cell neurite extension. *J. Biol. Chem.* **278**, 35394-35402 (2003).
