# Supplementary material for: Structure-Guided Design of Selective Epac1 and Epac2 Agonists
Source: PLoS Biol. 2015 Jan 20;13(1):e1002038. doi: 10.1371/journal.pbio.1002038 (PMC4300089; doi:10.1371/journal.pbio.1002038)
Supplement: S1 Text — 31P, 13C, and 1H spectra of D-002, L-027, S-030, S-031, S-140, S-150, S-220, S-222, S-223, and S-280 are shown and tentative assignments are presented. (PDF) [file pbio.1002038.s006.pdf]

NMR spectra of representative cAMP analogues

D-002 2
L-027 4
S-030 6
S-031 8
S-140 10
S-150 12
S-220 14
S-222 16
S-223 18
S-280 20

D-002

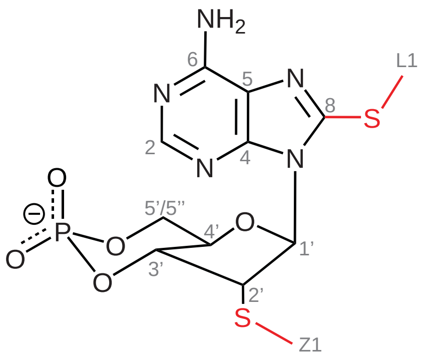

atom[1]	^{31}P /ppm[2]	^{13}C /ppm[2]	^{1}H /ppm[2]	multi-plicity[3]	J/Hz
P	-1.820				
2		154.59	8.123	s	
4 / 5 / 6 / 8		156.26 / 154.59 / 154.21 / 153.16			
1'		93.38	6.224	d	3.2
2'		52.03	4.056	dd	3.3 / 8.2
3'		80.36	5.419	t(d)	9.0 (1.5)
4'		76.16	4.134	dt	4.8 / 10.1
5'		69.38	4.463	ddd	4.8 / 9.7 / 21.0
5"			4.350	t(d)	10.1 / 1.5
L1 / Z1		17.18 / 16.71	2.701 / 2.155	s<br>s	

[1] annotation of C and H based on their position as indicated in the chemical structure
[2] signal position
[3] d, doublet; dd, double doublet; ddd, double double doublet; dt, double triplet; p, pentet; s, singlet; t, triplet; td, triple doublet

Spectra (see next page)

A. ^{13}P spectrum

B. ^{13}C spectrum

C. Water-suppressed ^{1}H spectrum

C1-C2. Magnifications of the spectrum shown in C. Note: The Y-axis varies between panels.

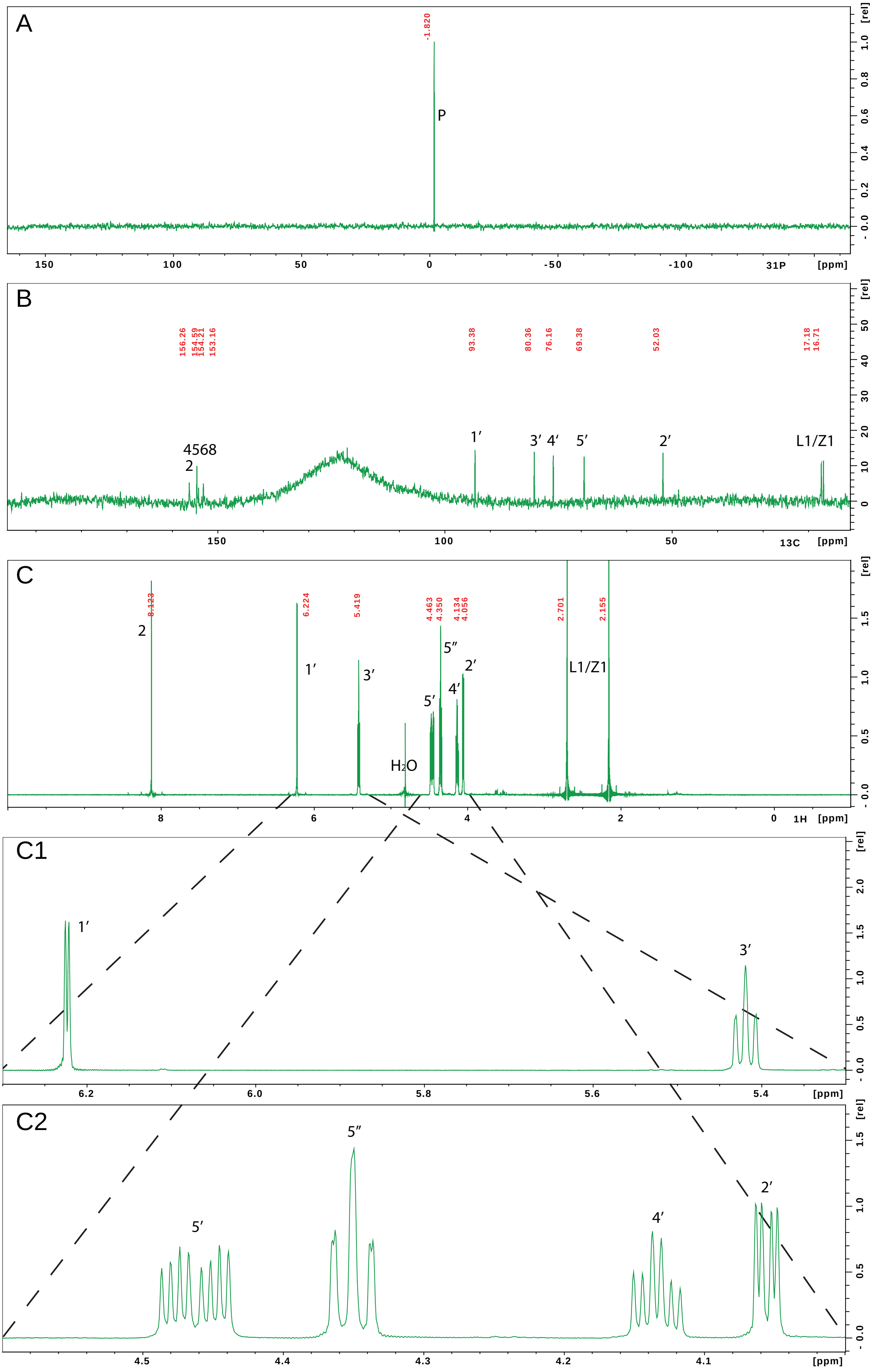
A
-1.820
P
[rel]
31P [ppm]
B
156.26
154.59
154.21
153.16
93.38
80.36
76.16
69.38
52.03
17.18
16.71
4568
2
1′
3′ 4′
5′
2′
L1/Z1
13C [ppm]
C
8.123
6.224
5.419
4.463
4.350
4.134
4.056
2.701
2.155
2
1′
3′
H2O
5′
5″
4′
2′
L1/Z1
1H [ppm]
C1
1′
3′
[ppm]
C2
5′
5″
4′
2′
[ppm]

L-027

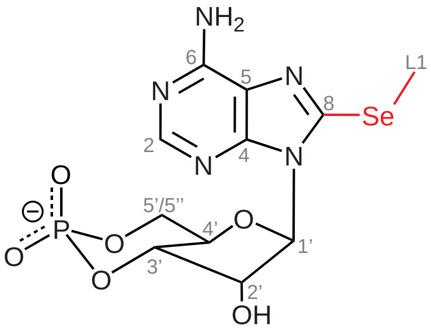

atom[1]	^{31}P /ppm[2]	^{13}C /ppm[2]	^{1}H /ppm[2]	multiplicity[3]	J/Hz
P	-1.472				
2		153.09	8.148	s	
4 / 5 / 6 / 8		155.25 / 153.32 / 153.09 / 148.94			
1'		95.98	6.077	s	
2'		74.41	4.794	dd	1.4 / 5.6
3'		79.54	5.129	ddd	2.0 / 5.9 / 10.0
4'		74.82	4.222	dt	4.8 / 10.3
5'		69.65	4.350	ddd	4.9 / 9.7 / 21.4
5"			4.136	dt	4.8 / 10.3
L1		10.58	2.559	s	

[1] annotation of C and H based on their position as indicated in the chemical structure
[2] signal position
[3] d, doublet; dd, double doublet; ddd, double double doublet; dt, double triplet; s, singlet;

Spectra (see next page)

A. ^{13}P spectrum

B. ^{13}C spectrum

C. Water-suppressed ^{1}H spectrum

C1. Magnifications of the spectrum shown in C.

C2. Magnifications of the same region as shown in C1 from a spectrum without water-suppression.

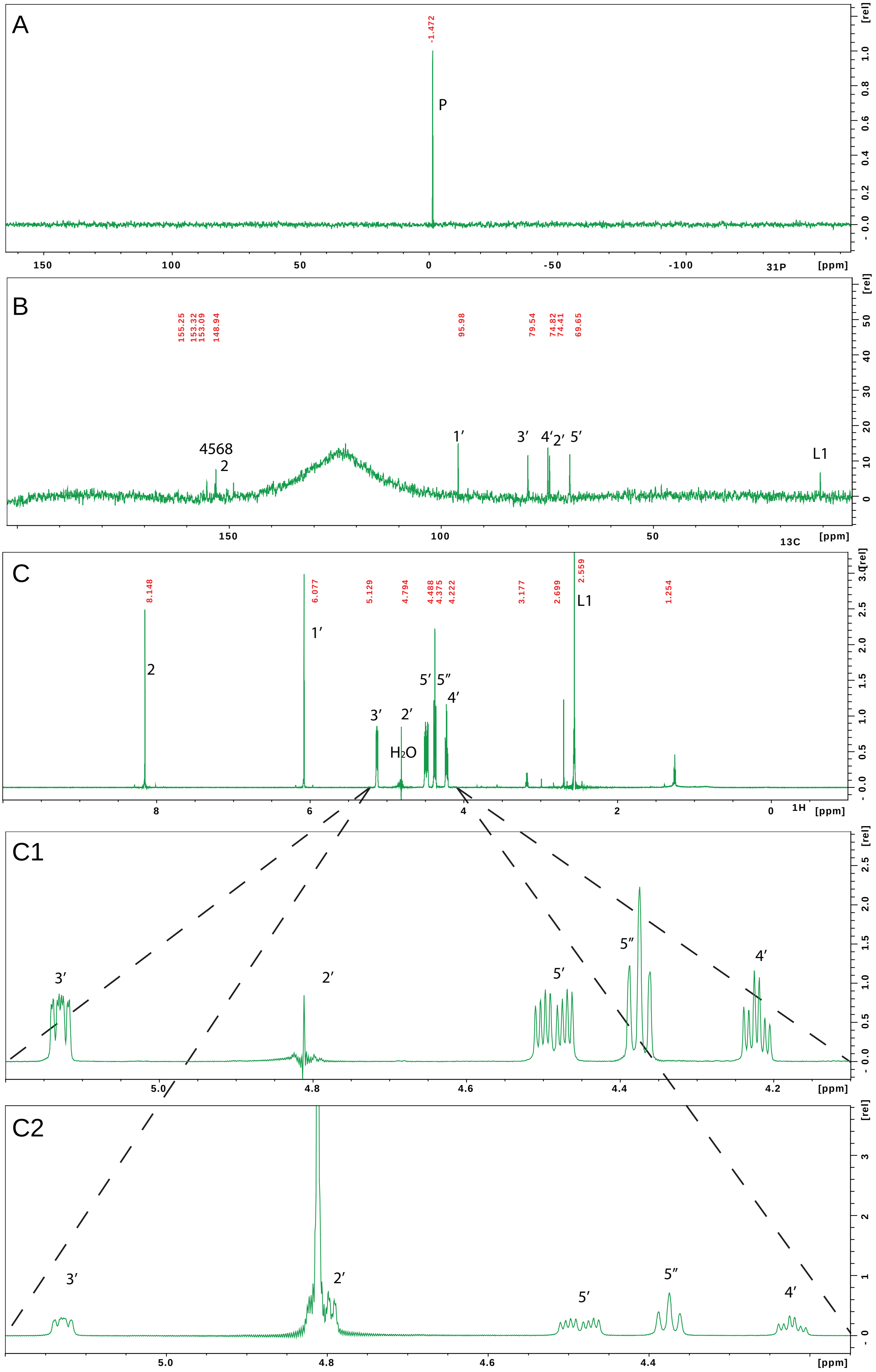

A
-1.472
P
31P [ppm]
[rel]
B
155.25 153.32 153.09 148.94
95.98
79.54 74.82 74.41 69.65
4568
2
1′
3′ 4′ 2′ 5′
L1
13C [ppm]
C
8.148
6.077
5.129
4.794
4.488 4.375 4.222
3.177
2.699
2.559
1.254
L1
1′
2
5′ 5″
4′
3′ 2′
H2O
1H [ppm]
C1
3′
2′
5′
5″
4′
C2
3′
2′
5′
5″
4′

S-030

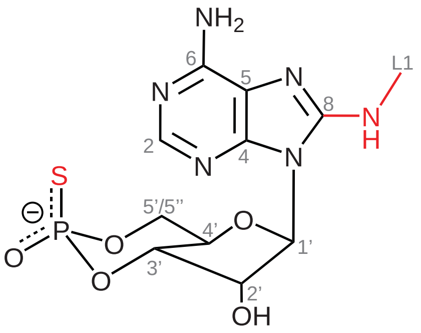

atom[1]	^{31}P /ppm[2]	^{13}C /ppm[2]	^{1}H /ppm[2]	multi-plicity[3]	J/Hz
P	54.506				
2		152.43	8.036	s	
4 / 5 / 6 / 8		156.17 / 154.31 / 152.44 / 152.12			
1'		93.90	5.814	s	
2'		73.37	5.000	d	5.6
3'		79.68	5.238	m	5.0
4'		74.14	4.151	dt	4.7 / 10.0
5'		70.11	4.436	ddd	4.7 / 9.6 / 24.0
5"			4.338	dt	2.6 / 10.0
L1		31.51	2.956	s	

[1] annotation of C and H based on their position as indicated in the chemical structure
[2] signal position
[3] d, doublet; ddd, double double doublet; dt, double triplet; m, multiplet; s, singlet; t, triplet

Spectra (see next page)

A. ^{13}P spectrum

B. ^{13}C spectrum

C. Water-suppressed ^{1}H spectrum

C1-C2. Magnifications of the spectrum shown in C. Note: The Y-axis varies between panels.

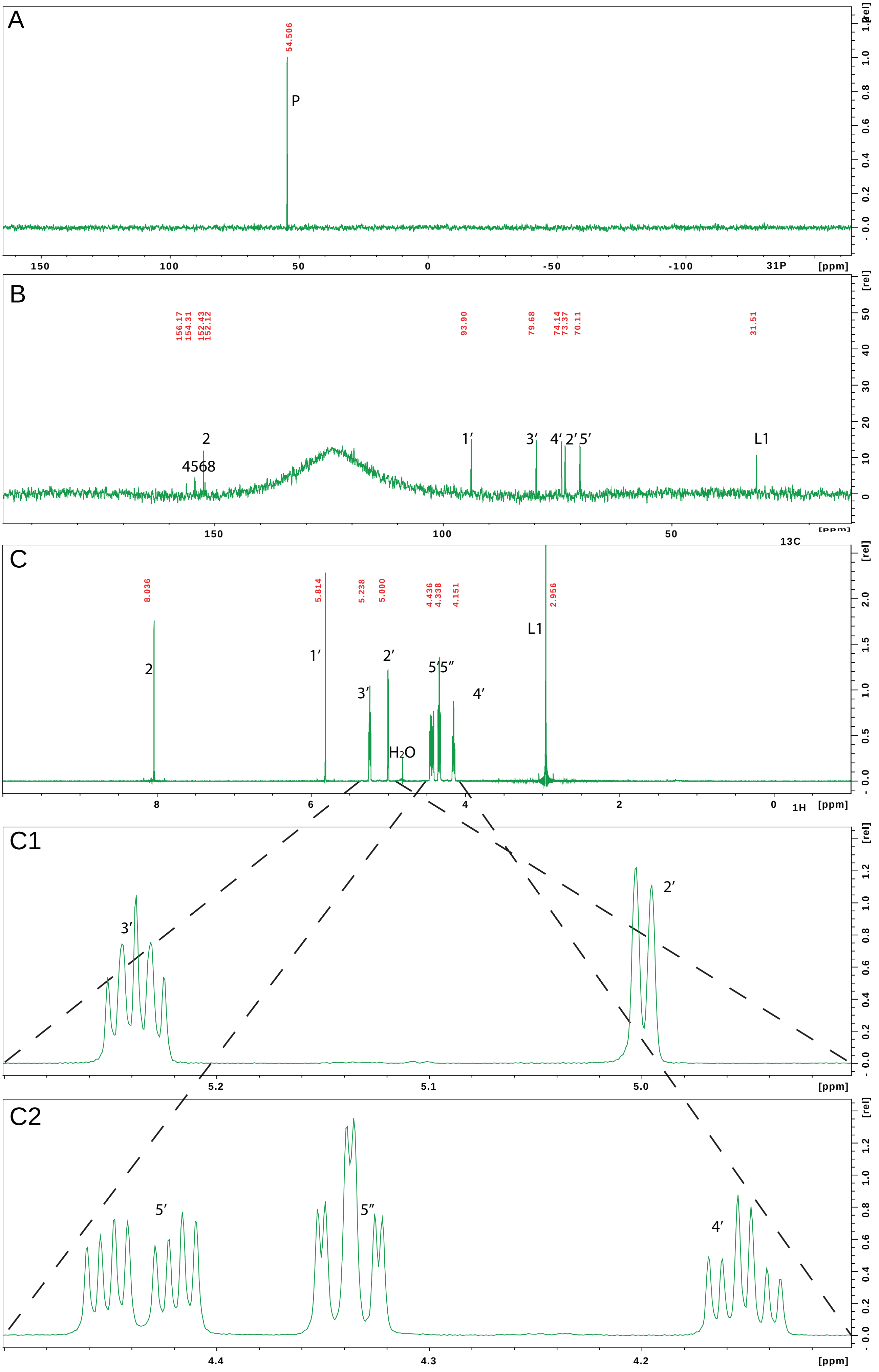
A
54.506
P
31P
[ppm]
[rel]
B
156.17
154.31
152.43
152.12
93.90
79.68
74.14
73.37
70.11
31.51
2
4568
1′
3′
4′
2′
5′
L1
13C
C
8.036
5.814
5.238
5.000
4.436
4.338
4.151
2.956
2
1′
2′
3′
5′5″
4′
L1
H2O
1H
C1
3′
2′
C2
5′
5″
4′

S-031

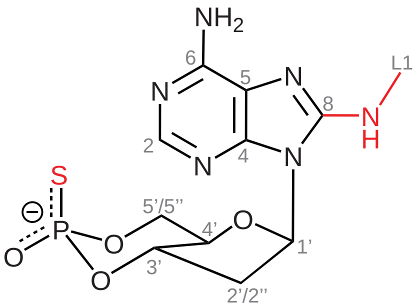

atom[1]	^{31}P /ppm[2]	^{13}C /ppm[2]	^{1}H /ppm[2]	multi-plicity[3]	J/Hz
P	53.715				
2		152.29	8.051	s	
4 / 5 / 6 / 8		156.33 / 154.26 / 152.29 / 152.02			
1'		85.20	6.224	dd	2.5 / 9.2
2'		35.5	2.938	m	2.8 / 7.9
2"			2.594	m	3.0 / 11.7
3'		79.25	5.348	m	3.7 / 14.0
4'		77.48	3.901	dt	4.6 / 10.0
5'		70.10	4.384	ddd	4.7 / 9.7 / 23.3
5"			4.339	dt	2.9 / 10.0
L1		31.56	2.960	s	

[1] annotation of C and H based on their position as indicated in the chemical structure
[2] signal position
[3] dd, double doublet; ddd, double double doublet; dt, double triplet; m, multiplet; s, singlet;

Spectra (see next page)

A. ^{13}P spectrum

B. ^{13}C spectrum

C. Water-suppressed ^{1}H spectrum

C1-C3. Magnifications of the spectrum shown in C. Note: The Y-axis varies between panels.

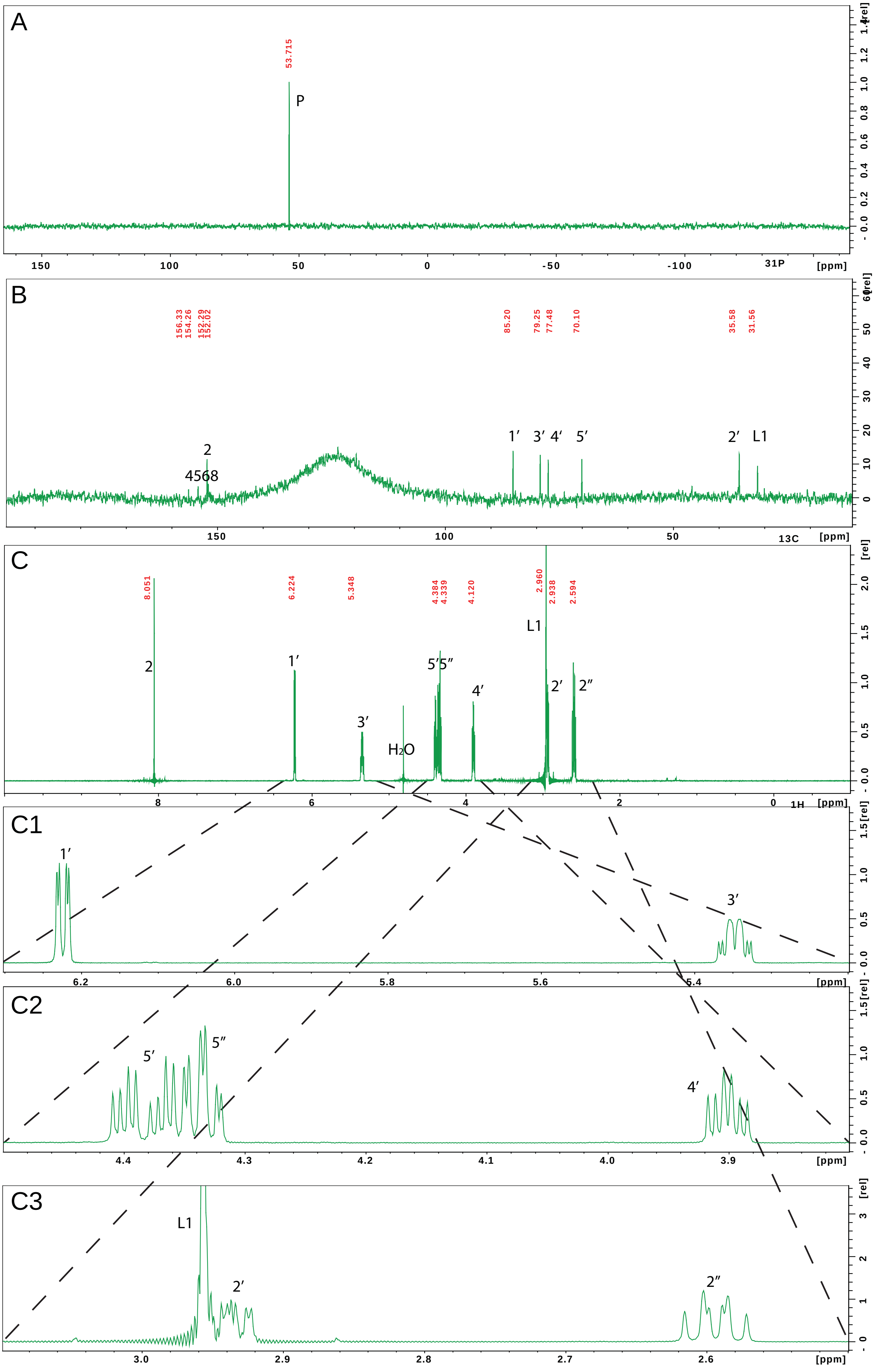
A
53.715
P
31P
[ppm]
[rel]
B
156.33
154.26
152.29
152.02
85.20
79.25
77.48
70.10
35.58
31.56
2
4568
1′
3′
4′
5′
2′
L1
13C
[ppm]
[rel]
C
8.051
6.224
5.348
4.384
4.339
4.120
2.960
2.938
2.594
2
1′
3′
H2O
5′5″
4′
L1
2′
2″
1H
[ppm]
[rel]
C1
1′
3′
[ppm]
[rel]
C2
5′
5″
4′
[ppm]
[rel]
C3
L1
2′
2″
[ppm]
[rel]

S-140

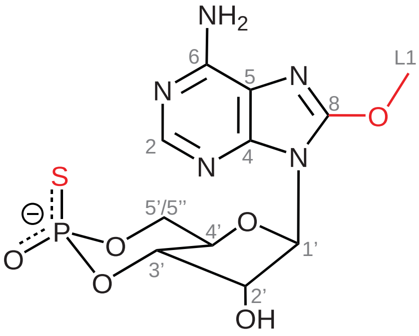

atom[1]	^{31}P /ppm[2]	^{13}C /ppm[2]	^{1}H /ppm[2]	multiplicity[3]	J/Hz
P	54.51				
2		154.50	8.131	s	
4 / 5 / 6 / 8		157.64 / 156.04 / 154.12 / 151.33			
1'		92.50	6.032	s	
2'		73.90	4.838	d	5.5
3'		79.75	5.145	m	5.0
4'		74.24	4.176	dt	4.8 / 10.4
5'		70.33	4.445	ddd	4.7 / 9.6 / 10.4
5"			4.321	dt	2.7 / 10.1
L1		60.69	4.201	s	

[1] annotation of C and H based on their position as indicated in the chemical structure
[2] signal position
[3] d, doublet; ddd, double double doublet; dt, double triplet; m, multiplet; s, singlet

Spectra (see next page)

A. ^{13}P spectrum

B. ^{13}C spectrum

C. Water-suppressed ^{1}H spectrum

C1-C2. Magnifications of the spectrum shown in C.

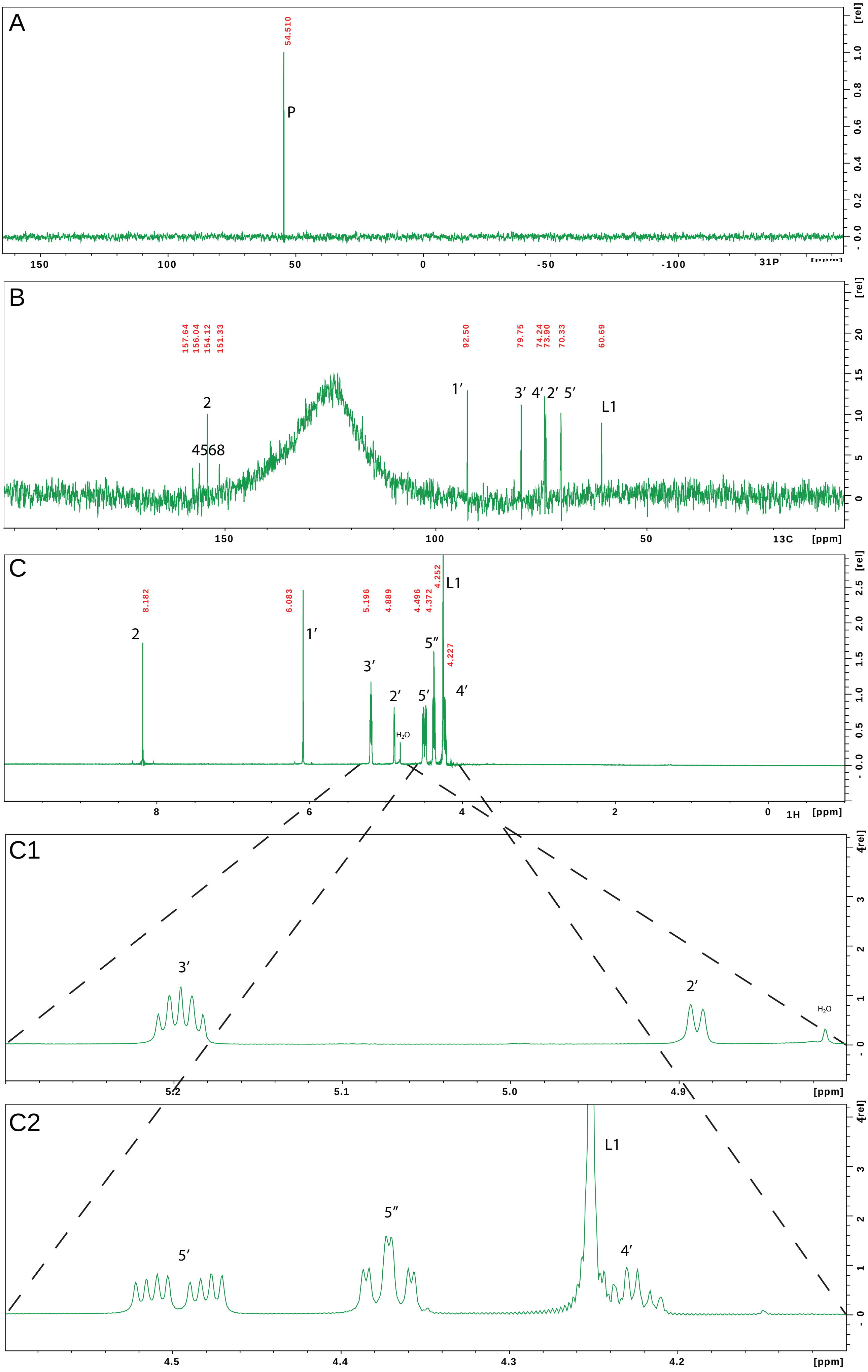
A
54.510
P
31P
[ppm]
[rel]
B
157.64
156.04
154.12
151.33
92.50
79.75
74.24
73.90
70.33
60.69
2
4568
1′
3′
4′
2′
5′
L1
13C
[ppm]
[rel]
C
8.182
6.083
5.196
4.889
4.496
4.372
4.252
4,227
2
1′
3′
2′
5′
5″
4′
L1
H2O
1H
[ppm]
[rel]
C1
3′
2′
H2O
[ppm]
[rel]
C2
5′
5″
L1
4′
[ppm]
[rel]

S-150

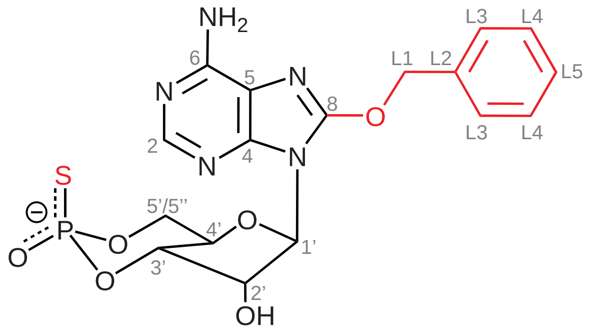

atom[1]	^{31}P /ppm[2]	^{13}C /ppm[2]	^{1}H /ppm[2]	multi-plicity[3]	J/Hz
P	54.732				
2		153.937	8.077	s	
4 / 5 / 6 / 8		156.59 / 155.98 / 153.97 / 151.09			
1'		92.41	6.005	s	
2'		73.78	4.756	d	5.5
3'		79.56	4.880	m	5.4
4'		74.28	4.084	dt	4.5 / 10.2
5'		70.03	4.350	ddd	4.2 / 9.3 / 24.0
5''			4.136	dt	2.0 / 10.5
L1		75.70	5.521	dd	11.8 / 77.3
L2		136.90			
L3		131.48	7.531	d	7.4
L4		131.81	7.429	t	7.5
L5		131.91	7.388	t	7.5

[1] annotation of C and H based on their position as indicated in the chemical structure
[2] signal position
[3] d, doublet; dd, double doublet; ddd, double double doublet; dt, double triplet; m, multiplet; s, singlet; t, triplet

Spectra (see next page)

A. ^{13}P spectrum

B. ^{13}C spectrum

C. Water-suppressed ^{1}H spectrum. Insert: Overlay of the water-suppressed ^{1}H spectrum (red) with a non-supressed spectrum (green) in the region of the water peak.

C1-C3. Magnifications of the spectrum shown in C. Note: The Y-axis varies between panels.

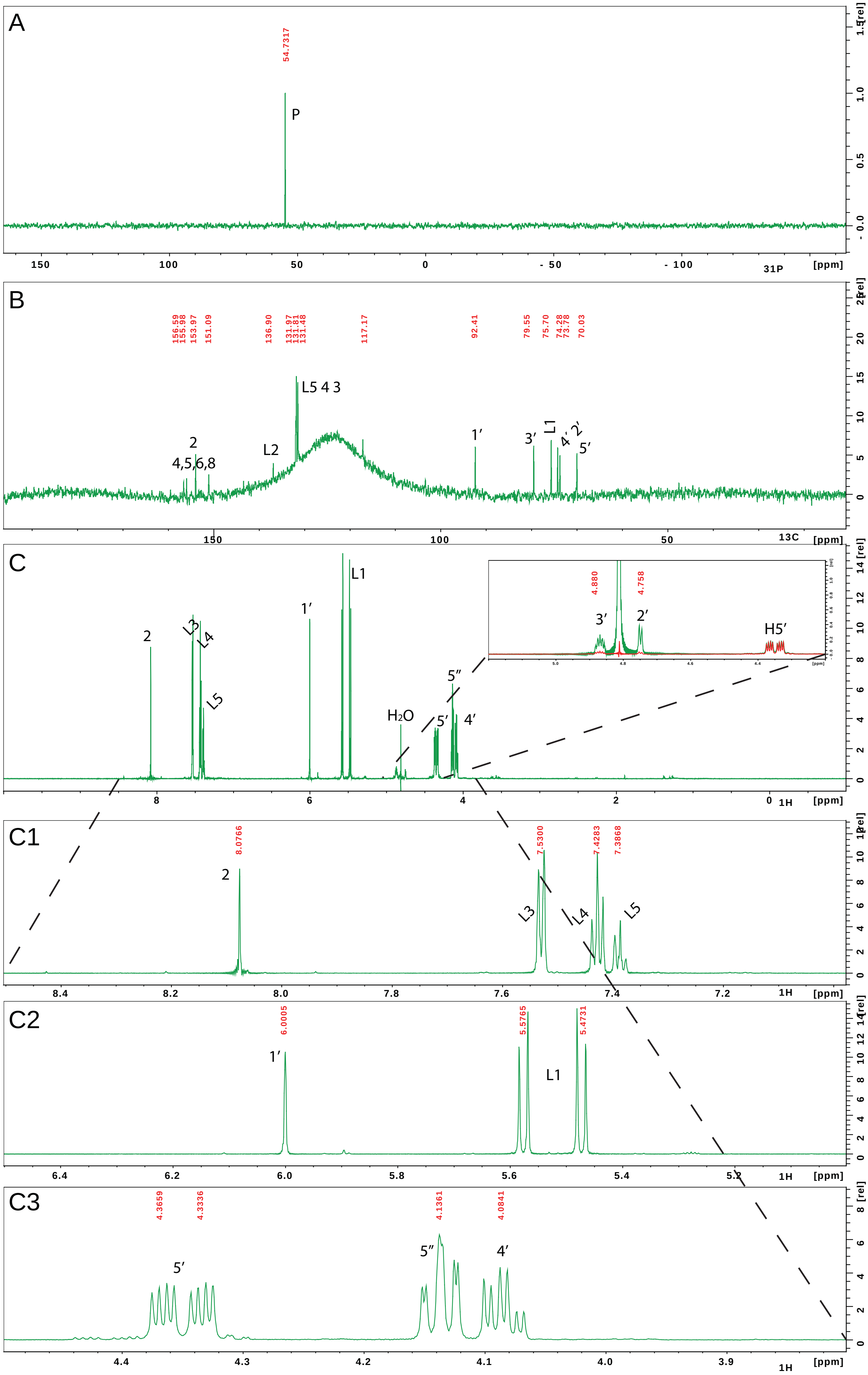
A
54.7317
P
31P
[ppm]
B
156.59 155.98 153.97 151.09 136.90 131.97 131.81 131.48 117.17 92.41 79.55 75.70 74.28 73.78 70.03
L5 4 3
2
4,5,6,8
L2
1′
3′
L1
4′
2′
5′
13C
C
L1
1′
2
L3
L4
L5
H2O
5″
5′
4′
4.880
4.758
3′
2′
H5′
1H
C1
8.0766
7.5300
7.4283
7.3868
C2
6.0005
5.5765
5.4731
C3
4.3659
4.3336
4.1361
4.0841
5″
[rel]

S-220

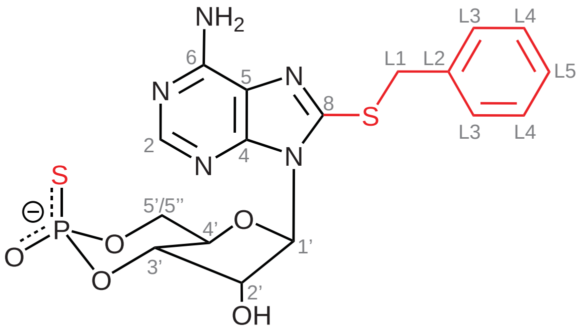

atom[1]	^{31}P /ppm[2]	^{13}C /ppm[2]	^{1}H /ppm[2]	multi-plicity[3]	J/Hz
P	54.808				
2		155.218	8.087	s	
4 / 5 / 6 / 8		155.683 / 155.156 / 152.648 / 151.013			
1'		94.752	6.023	s	
2'		74.36	4.248	d	5.5
3'		79.21	5.321	m	5.0
4'		74.65	4.084	dt	5.7 / 10.0
5'		69.8	4.366	m	5.6 / 9.7
5"			4.366	m	5.6 / 9.7
L1		41.039	4.367	dd	13.3 / 18.3
L2		139.205			
L3		131.505	7.28	d	6.7
L4		131.505	7.236	m	6.8
L5		130.57	7.205	m	

[1] annotation of C and H based on their position as indicated in the chemical structure
[2] signal position
[3] d, doublet; dd, double doublet; dt, double triplet; m, multiplet; s, singlet;

Spectra (see next page)

A. ^{13}P spectrum

B. ^{13}C spectrum

C. Water-suppressed ^{1}H spectrum

C1-C2. Magnifications of the spectrum shown in C.

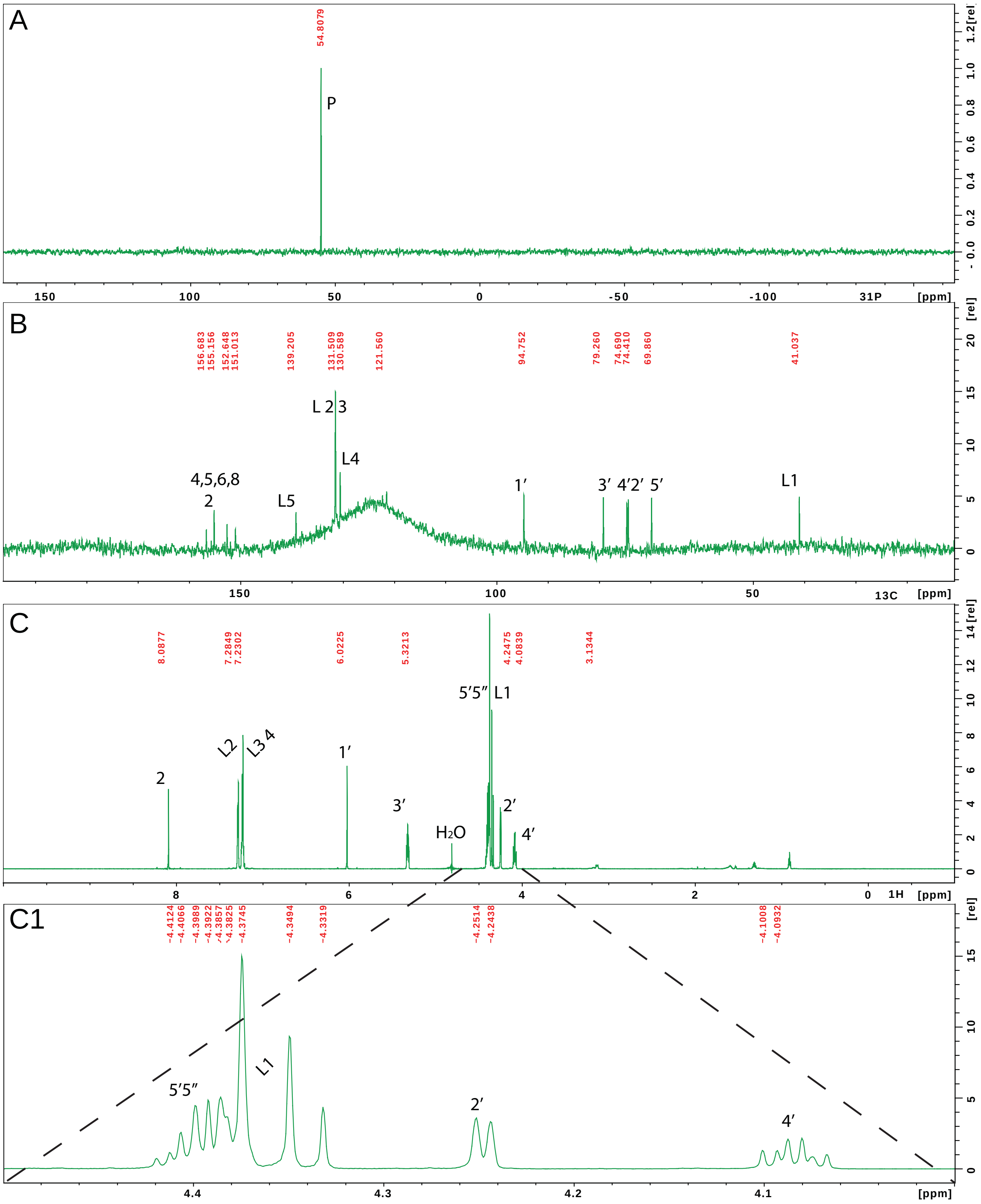
A
54.8079
P
31P [ppm]
B
156.683
155.156
152.648
151.013
139.205
131.509
130.589
121.560
94.752
79.260
74.690
74.410
69.860
41.037
L 2 3
L4
4,5,6,8
2
L5
1′
3′ 4′2′ 5′
L1
13C [ppm]
C
8.0877
7.2849
7.2302
6.0225
5.3213
4.2475
4.0839
3.1344
5′5″ L1
L2
L3 4
1′
2
3′
H2O
2′
4′
1H [ppm]
C1
4.4124
4.4066
4.3989
4.3922
4.3857
4.3825
4.3745
4.3494
4.3319
4.2514
4.2438
4.1008
4.0932
5′5″
L1
2′
4′
[ppm]

S-222

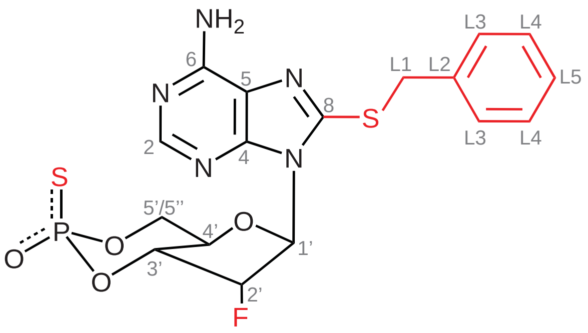

atom[1]	^{31}P /ppm[2]	^{13}C /ppm[2]			^{1}H /ppm[2]	multi-plicity[3]	J/Hz
P	54.763						
2		155.28			8.082	s	
4 / 5 / 6 / 8		156.70 / 155.28 / 152.62 / 151.03					
1'		91.82	d	36.52	6.236	d	24.9
2'		93.65	d	189.90	5.178	dd	4.6 / 55.11
3'		78.36			5.449	m	4.4 / 5.0 / 27.7
4'		74.19			4.120	dt	5.0 / 10.3
5'		69.74			4.400	ddd	5.1 / 9.8 / 23.9
5''					4.360	dt	2.8 / 9.8
L1		41.05			4.341	m	4.9
L2		139.07					
L3		131.51			7.263	dd	1.7 / 7.8
L4		131.51			7.218		
L5		130.62			7.218	m	

[1] annotation of C and H based on their position as indicated in the chemical structure
[2] signal position
[3] d, doublet; dd, double doublet; ddd double double doublet ; dt, double triplet; m, multiplet; s, singlet;

Spectra (see next page)

A. ^{13}P spectrum

B. ^{13}C spectrum

C. Water-suppressed ^{1}H spectrum.

C1-C3. Magnifications of the spectrum shown in C. Note: The Y-axis varies between panels.

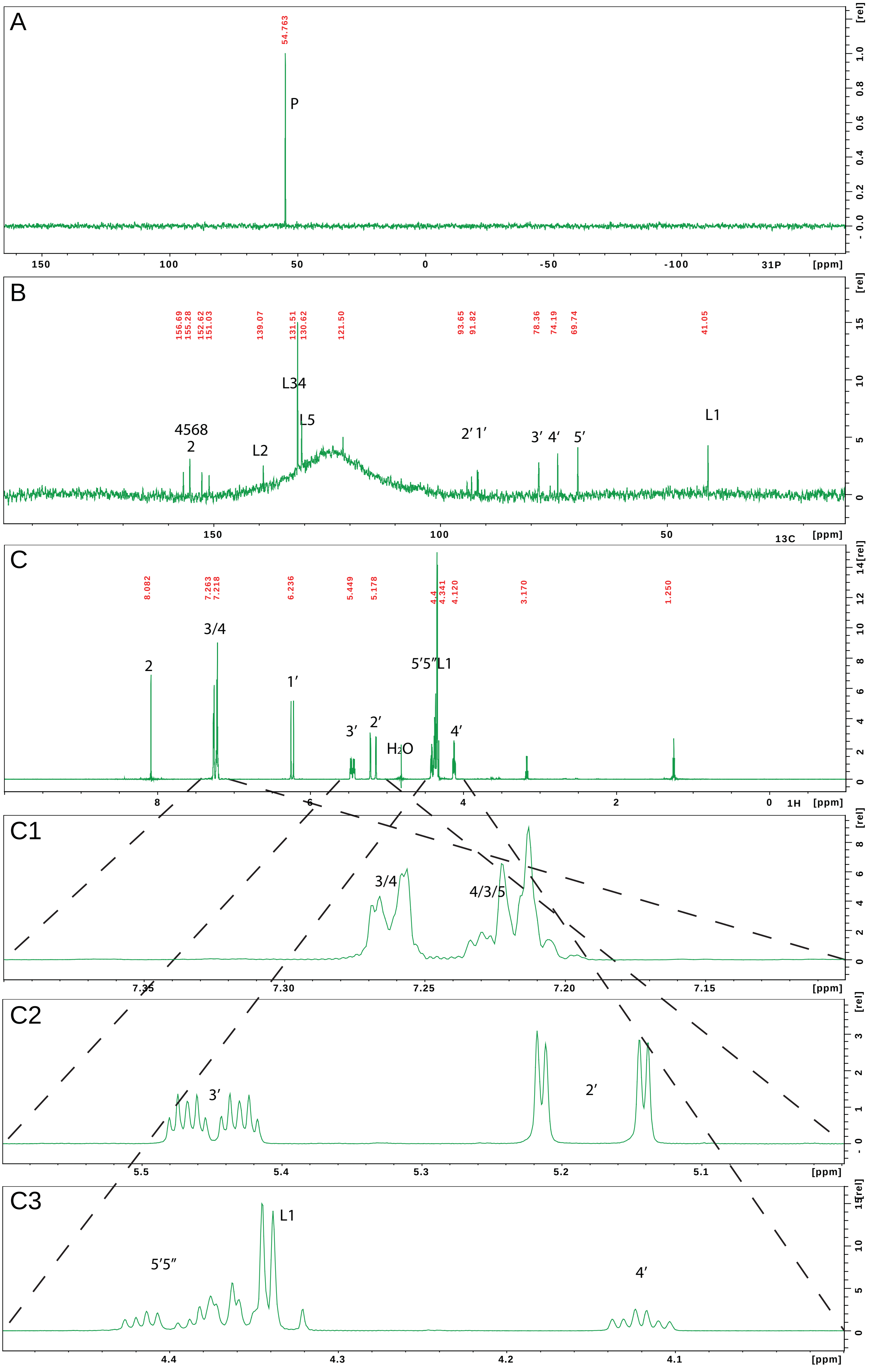
A
54.763
P
31P
[ppm]
[rel]
B
156.69
155.28
152.62
151.03
139.07
131.51
130.62
121.50
93.65
91.82
78.36
74.19
69.74
41.05
L34
L5
4568
2
L2
2′ 1′
3′ 4′
5′
L1
13C
C
8.082
7.263
7.218
6.236
5.449
5.178
4.341
4.120
3.170
1.250
3/4
2
1′
3′
2′
H2O
5′5″L1
4′
1H
C1
3/4
4/3/5
C2
3′
2′
C3
L1
5′5″
4′

S-223

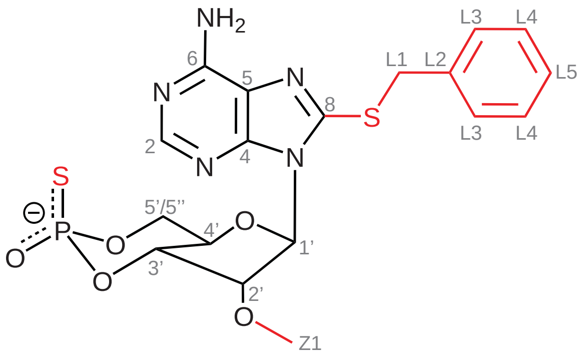

atom[1]	^{31}P /ppm[2]	^{13}C /ppm[2]	^{1}H /ppm[2]	multi-plicity[3]	J/Hz
P	54.658				
2		155.22	8.112	s	
4 / 5 / 6 / 8		156.76 / 155.22 / 152.45 / 150.75			
1'		92.68	6.050	s	
2'		83.33	3.678	d	5.9
3'		79.11	5.403	m	5.0 / 5.4
4'		75.05	4.004	m	5.0
5'		69.89	4.377	m	4.9 / 6.3
5''			4.377	m	4.9 / 6.3
L1		41.29	4.377	dd	13.3 / 43.0
L2		139.51			
L3		131.52	7.279	d	2.0 / 7.4
L4		131.52	7.225	m	2.0 / 4.7
L5		130.66	7.225	m	
Z1		60.88	3.248	s	

[1] annotation of C and H based on their position as indicated in the chemical structure
[2] signal position
[3] d, doublet; dd, double doublet; m, multiplet; s, singlet;

Spectra (see next page)

A. ^{13}P spectrum

B. ^{13}C spectrum

C. Water-suppressed ^{1}H spectrum.

C1-C2. Magnifications of the spectrum shown in C. Note: The Y-axis varies between panels.

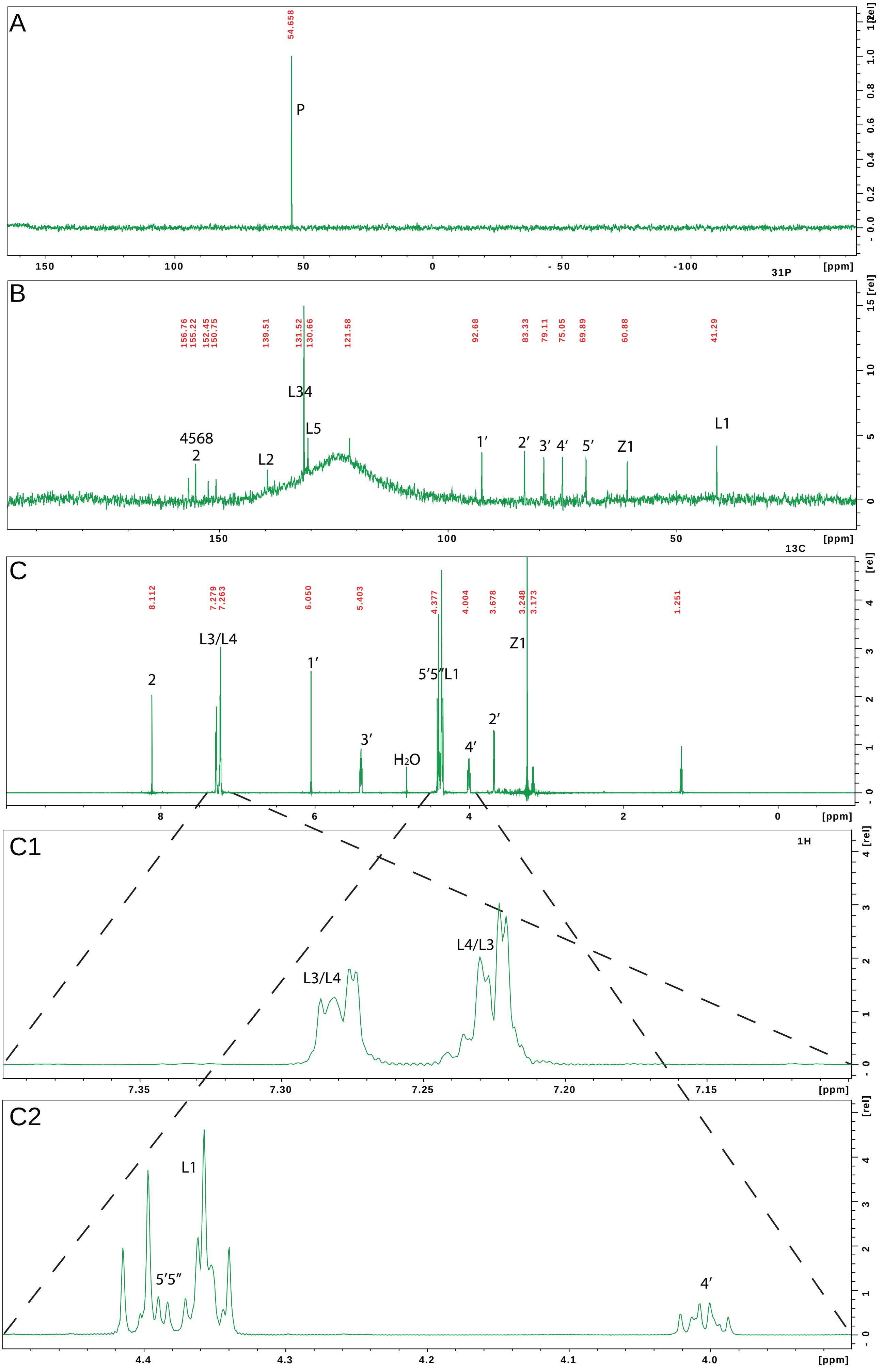
A
54.658
P
[rel]
150
100
50
0
- 50
-100
[ppm]
31P
B
156.76
155.22
152.45
150.75
139.51
131.52
130.66
121.58
92.68
83.33
79.11
75.05
69.89
60.88
41.29
L34
L5
4568
2
L2
1′
2′
3′
4′
5′
Z1
L1
[rel]
150
100
50
[ppm]
13C
C
8.112
7.279
7.263
6.050
5.403
4.377
4.004
3.678
3.248
3.173
1.251
L3/L4
2
1′
5′5″L1
Z1
2′
3′
4′
H2O
[rel]
8
6
4
2
0
[ppm]
C1
1H
L3/L4
L4/L3
[rel]
7.35
7.30
7.25
7.20
7.15
[ppm]
C2
L1
5′5″
4′
[rel]
4.4
4.3
4.2
4.1
4.0
[ppm]

S-280

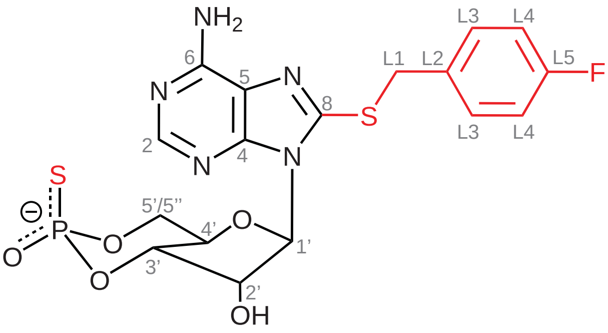

atom[1]	^{31}P /ppm[2]	^{13}C /ppm[2]	^{1}H /ppm[2]	multi-plicity[3]	J / Hz
P	54.817				
2		155.19	8.090	s	
4 / 5 / 6 / 8		156.69 / 155.19 / 152.65 / 150.90			
1'		94.76	6.020	s	
2'		74.45			
3'		79.29	5.325	m	5.0
4'		74.69	4.093	m	5.7 / 10.2
5'		69.85	4.402	ddd	5.0 / 9.6 / 22.3
5"			4.369	dd	4.9 / 17.9
L1		40.29	4.333	dd	5.0 / 9.6 / 22.3
L2		133.38			
L3 / L4		133.38	7.261	d	5.0 / 9.6 / 22.3
L4 / L3		135.09	6.938	t	4.9 / 17.9
L5		118.1 d 21			

[1] annotation of C and H based on their position as indicated in the chemical structure
[2] signal position
[3] d, doublet; dd, double doublet; ddd, double double doublet; m, multiplet; s, singlet; t, triplet

Spectra (see next page)

A. ^{13}P spectrum

B. ^{13}C spectrum

C. Water-suppressed ^{1}H spectrum.

C1-C2. Magnifications of the spectrum shown in C. Note: The Y-axis varies between panels.

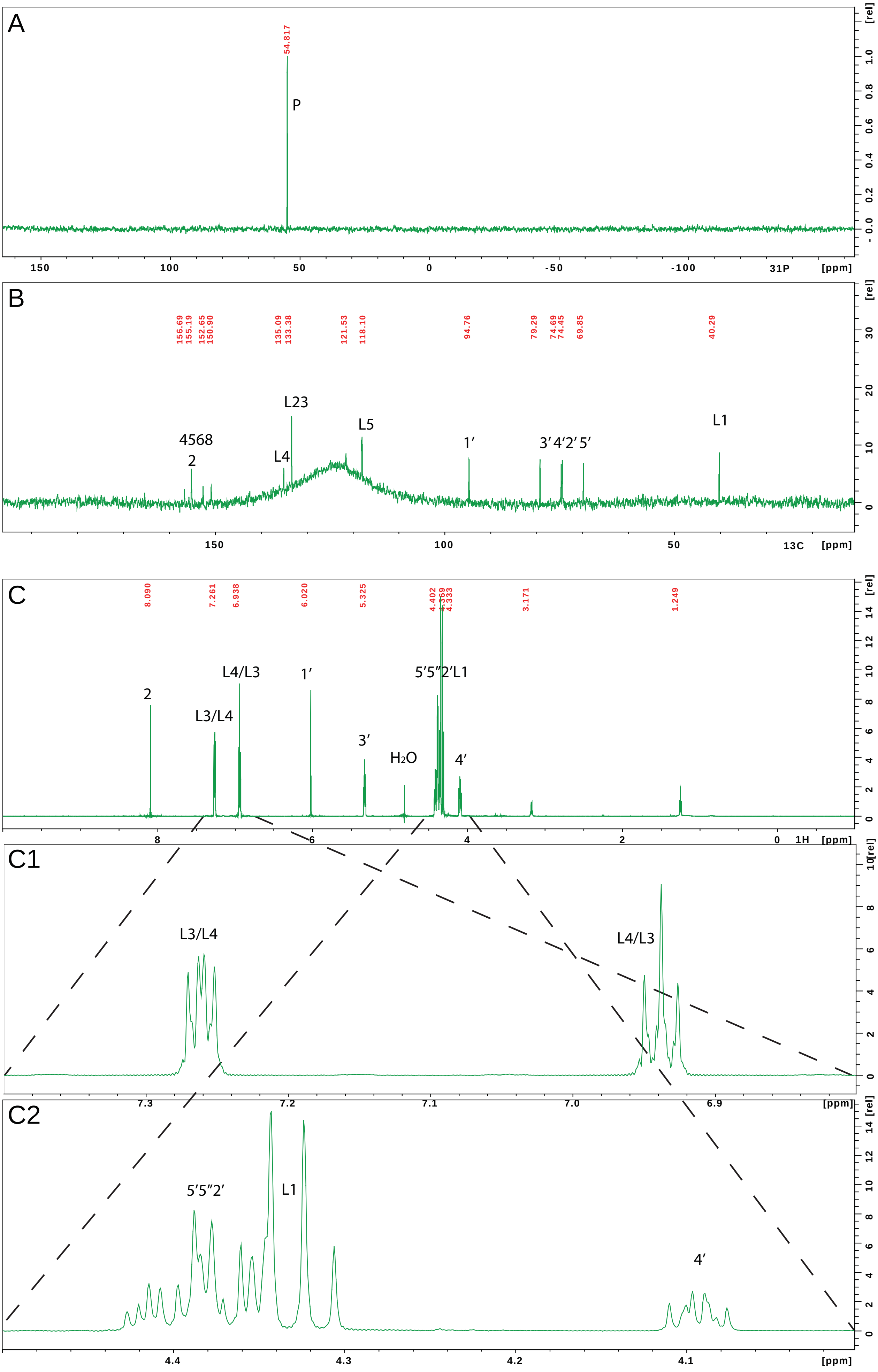
A
54.817
P
[rel]
31P [ppm]
B
156.69
155.19
152.65
150.90
135.09
133.38
121.53
118.10
94.76
79.29
74.69
74.45
69.85
40.29
4568
2
L23
L4
L5
1′
3′4′2′5′
L1
13C [ppm]
C
8.090
7.261
6.938
6.020
5.325
4.402
4.369
4.333
3.171
1.249
2
L3/L4
L4/L3
1′
3′
H2O
5′5″2′L1
4′
1H [ppm]
C1
L3/L4
L4/L3
[ppm]
C2
5′5″2′
L1
4′
[ppm]
